# The impact of Charcot-Leyden Crystal protein on mesothelioma chemotherapy: targeting eosinophils for enhanced chemosensitivity

**DOI:** 10.1016/j.ebiom.2024.105418

**Published:** 2024-10-30

**Authors:** Mégane Willems, Malik Hamaidia, Alexis Fontaine, Mélanie Grégoire, Louise Halkin, Lea Vilanova Mañá, Roxane Terres, Majeed Jamakhani, Sophie Deshayes, Yves Brostaux, Vincent Heinen, Renaud Louis, Bernard Duysinx, Didier Jean, Eric Wasielewski, Arnaud Scherpereel, Christophe Blanquart, Luc Willems

**Affiliations:** a Molecular and Cellular Epigenetics (GIGA) and Molecular Biology (TERRA), University of Liege, Liege & Gembloux, Belgium; b Institut National de la Santé et de la Recherche Médicale (INSERM) U1232 Centre de Recherche en Cancérologie et Immunologie Nantes Angers (CRCINA), Nantes, France; c Modelisation and development, Gembloux Agro-Bio Tech, University of Liege, Gembloux, Belgium; d Department of Pneumology (University Hospital of Liege), Liege, Belgium; e Centre de Recherche des Cordeliers (INSERM), Sorbonne Université (Université de Paris), Functional Genomics of Solid Tumors, Paris, France; f Department of Pneumology and Thoracic Oncology (CHU Lille) and INSERM U1189 (ONCOTHAI), Lille, France

**Keywords:** Mesothelioma, Charcot-leyden crystal protein, Eosinophils, Chemoresistance

## Abstract

**Background:**

In mesothelioma (MPM), clinical evidence indicates that the absolute eosinophil count negatively correlates with overall survival and response to standard chemotherapy. Since eosinophils poorly infiltrate MPM tumours, we hypothesised that endocrine rather than paracrine pathways mediate the therapeutic response. We thus studied the effect of eosinophil-associated factors on response to chemotherapy in mesothelioma.

**Methods:**

The culture supernatant conditioned by primary human eosinophils was added to mesothelioma cells in presence of the standard chemotherapeutic regimen. The effectiveness of an anti-eosinophil treatment was evaluated in a preclinical model of C57BL/6 mice transplanted with mesothelioma tumour cells.

**Findings:**

Supernatant of eosinophils differentiated from EOL1 cells or directly isolated from peripheral blood inhibited apoptosis induced by cisplatin and pemetrexed in 2D cultures and in spheroids. Transcriptomic analysis indicated that the anti-apoptotic effect mediated by eosinophils involved molecular interactions with the Charcot-Leyden Crystal protein or Galectin-10 (CLC-P/Gal10). The functional relevance of CLC-P/Gal10 was demonstrated by antibody-mediated depletion. Recombinant human CLC-P/Gal10 mimicked the anti-apoptotic activity of eosinophil-derived supernatants. In the mouse model, eosinophilia did not significantly affect tumour growth but altered the response to chemotherapy. Finally, pretreatment of eosinophilia with the anti-Siglec-F antibody before chemotherapy restored the effectiveness of the treatment.

**Interpretation:**

This study provides a mechanistic rationale to clinical evidence correlating the poor outcome of patients with mesothelioma and with eosinophil-derived CLC-P/Gal10, opening new prospects for intervention in this fatal solid tumour.

**Funding:**

Belgian Foundation against Cancer, Fonds National de la Recherche Scientifique (FNRS), Télévie, Foundation Léon Fredericq, ULiège.

**Research in context**

**Evidence before this study**

Clinical evidence indicates that the absolute eosinophil counts (AEC) in peripheral blood correlate with poorer overall survival in mesothelioma. A retrospective study of 242 patients with histologically proven mesothelioma, conducted across three centres, reveals a significant impact on the response to standard chemotherapy within the AEC ≥ 220/μL subset.

**Added value of this study**

This report underscores a crucial mechanism connecting eosinophils to a diminished response to chemotherapy. Eosinophil-derived Charcot-Leyden crystal protein/galectin-10 (CLC-P/Gal10) mediates chemoresistance by interacting with mesothelioma tumour cells. Additionally, the levels of CLC-P/Gal10 in plasma and pleural fluids correlate with poorer overall survival. Finally, preclinical evidence from a mouse model demonstrates that an anti-eosinophil treatment restores chemosensitivity.

**Implications of all the available evidence**

Understanding the fundamental mechanism linking eosinophils to a suboptimal response to chemotherapy provides direct opportunities for improved treatments in mesothelioma. Given the ready availability of anti-eosinophilic treatments, the findings of this study can be directly evaluated for potential integration into future therapeutic practices.

## Introduction

Malignant mesothelioma is an aggressive cancer that is mainly induced by chronic inflammation and oxidative stress caused by inhaled asbestos fibres. The disease affects serous membranes of the pleura, the peritoneum, the pericardium and, less frequently, the tunica vaginalis testis.^1^ According to the histology, pleural mesothelioma (MPM) can be classified as epithelioid (the most frequent subtype, accounting for 60–80% of cases), sarcomatoid (∼10%) and biphasic (10–15%). Despite the progressive ban of asbestos use in most countries, incidence of MPM is still increasing worldwide.^2^^,^^3^ In principle, MPM patients may be eligible for a standard multimodal treatment including surgery, radiotherapy and/or chemotherapy.^4^^,^^5^ Since 2003, the standard-of-care for unresectable MPM has been the combination of a DNA cross-linking agent (cisplatin or carboplatin) and an antifolate (pemetrexed).^4^ With an increase of median overall survival (OS) from 9.1 to 13–16 months, the benefit of this treatment remains nevertheless modest.^4^ Combination of an anti-VEGF antibody (bevacizumab) to the cisplatin/pemetrexed regimen slightly improves the median OS up to 18.8 months (*vs* 16.0 months in the control arm).^5^ In addition, immune checkpoint inhibitors (ICIs) targeting PD-1 (nivolumab) and CTLA-4 (ipilimumab) are particularly effective in the sarcomatoid subset of MPM and are now incorporated in practice guidelines.^6^^,^^7^ Despite these recent improvements, the prognosis of MPM patients remains usually poor.

Increasing evidence indicates that the immune tumour microenvironment (TME) is a major parameter that orients the clinical outcome of cancer patients.^8^, ^9^, ^10^ Although MPM has been initially considered as a cold tumour,^11^^,^^12^ a significant proportion of cases are characterised by an infiltration of tumour-associated macrophages (TAMs) and Tregs.^10^^,^^13^, ^14^, ^15^, ^16^, ^17^, ^18^ The presence of these immunosuppressive cells in tumours or in the pleural fluid of patients with MPM correlates with poor therapeutic response and bad prognosis. Furthermore, TAMs and monocyte myeloid-derived immunosuppressive cells (M-MDSCs) constitute a major fraction of the TME.^19^^,^^20^ The selective depletion of myeloid cells in a knockout mouse model promotes tumour rejection, indicating the role of monocyte-derived TAMs in MPM development.^21^ In contrast, targeting tissue-resident large peritoneal/pleural macrophages abrogates the antitumoral memory immunity. Besides phagocytosis, cytokine production and antibody-dependent cell-mediated cytotoxicity (ADCC), macrophages also exert a cytotoxic activity by cell-to-cell contact with MPM cells. Consistently, tumoricidal macrophages exert immunoediting activity against mesothelioma tumours in the absence of adaptive immunity.^10^^,^^18^

It is widely accepted that macrophages are essential mediators of MPM tumour growth. However, the mechanisms underlying their role in MPM remain unknown. The current hypotheses are even complexified by their phenotypic diversity, their interplay with other immune cells and by the fact that immune cells may also not directly infiltrate the TME.^22^^,^^23^ The prognosis of MPM is indeed negatively influenced by systemic inflammation markers such as the C-reactive protein (CRP).^24^ In this context, we recently provided clinical evidence indicating that blood eosinophils inversely correlate with response to chemotherapy and patient's overall survival. Excess of absolute eosinophil counts in the peripheral blood prior to chemotherapy is associated with worse outcome and quicker relapse in MPM.^25^

By releasing cytokines, including chemokines, growth factors and enzymes, eosinophils mediate well-characterised immune-related mechanisms such as allergic disorders and pathogen infections.^26^, ^27^, ^28^ The role of eosinophils in tumour development is far from being well understood. Eosinophils produce both anti- (*e.g.,* TNF-α, granzyme, cationic proteins and IL-12) and pro-tumorigenic molecules (*e.g.,* TGF-β1, VEGF).^29^ In the TME, eosinophils interact with macrophages, dendritic cells, T-lymphocytes and mast cells.^29^, ^30^, ^31^ IL-4, IL-13 and IL-10 produced by eosinophils lead to the differentiation of macrophages into the M2-like TAM phenotype.^31^^,^^32^ Eosinophils also promote T-cell proliferation and activation via Th1 (*e.g.,* IFN-γ, IL-2 and IL-12) and Th2 (*e.g.,* IL-4, IL-5, IL-6, IL-10 and IL-13) cytokines.^26^^,^^33^ As antigen presenting cells (APCs), eosinophils can directly modulate the adaptive T cell response.^33^ Finally, eosinophils induce angiogenesis by promoting endothelial cell proliferation and by producing VEGF, FGF and PDGF.^29^^,^^34^ The functional complexity of eosinophils and their interplay with multiple immune cells likely explain their pro- and anti-tumorigenic effects, as well as their association with both good (*e.g*., melanoma and colorectal cancer) and poor prognosis (*e.g.,* Hodgkin's lymphoma, cervical carcinoma) depending on the cancer type.^32^

Based on the correlation observed in the retrospective analysis, the objectives of the present study are (i) to investigate the impact of eosinophils on response to therapy in cell culture and mouse models and (ii) to characterise the mechanisms involved and identify the factors that mediate eosinophil activity.

## Methods

### Differentiation of the EOL1 progenitor cell line into eosinophils

The EOL1 cell line (RRID:CVCL_0258) was cultured in RPMI-1640 (Lonza) supplemented with 10% heat-inactivated foetal bovine serum (FBS, Lonza), 1% penicillin and streptomycin (10,000 U/ml, Lonza), 1% sodium pyruvate (Gibco) and 1% amphotericin B (Gibco) (*i.e.,* RPMI_EOL1_) at 37 °C in a humidified atmosphere containing 5% CO_2_. EOL1 (2 × 10^5^ cells/well in a 24-well plate) were differentiated into eosinophil-like cells using 2 mM sodium valproate (Sigma) in RPMI_EOL1_ medium for 8 days. To reach further maturation, Dif-EOL1 were incubated with 100 ng/mL interleukin-5 (IL-5, ImmunoTools Cat #11340055) for 48 h. Cell culture supernatants from progenitors (EOL1) and Dif-EOL1 were collected, cleared by centrifugation, aliquoted and stored at −80 °C for further experiments.

To control for adequate differentiation, the ability of Dif-EOL1 to express CCR3, IL-5Rα, CD63 and CLC-P/Gal10 was determined by flow cytometry immunophenotyping and confocal microscopy (see M&M paragraph below). To quantify eosinophil peroxidase activiy, 5 × 10^5^ EOL1 and Dif-EOL1 cells were incubated in 500 μL of a substrate solution containing o-phenylenediamine (Sigma, OPD; 0.4 mM), Tris–HCl (Sigma, 0.4 M, pH 8.0) and H_2_O_2_ (1.25 × 10^−^^5^ v/v).^35^ After 30 min at room temperature in the dark and addition of an equal volume of HCl 4N, the optical density was determined at λ = 492 nm (SpectraMax Plus, Molecular Devices).

### Isolation of primary human eosinophils

To isolate primary human eosinophils, buffy coats were obtained from healthy donors (Red Cross of Belgium). The use of human samples was approved by the institutional ethic committee of the University Hospital (CHU, Sart-Tilman) under the reference #2012/8. Granulocytes were isolated by gradient centrifugation on lymphoprep (1.077 g/mL, Stemcell Technologies). Erythrocytes were lysed with RBC lysis buffer (BioLegend). Granulocytes (10 × 10^6^ cells) were washed in PBS before labelling with 1 μg/mL anti-CCR3 antibody (Invitrogen Cat# 14-1939-82, RRID:AB_795829) for 30 min at 4 °C. Eosinophils were purified by magnetic cell sorting using microbeads coupled with anti-mouse IgG (Miltenyi Biotec Cat# 130-048-402, RRID:AB_244361). CCR3^+^ primary human eosinophils were then cultured in RPMI supplemented with 1% sodium pyruvate at 37 °C for 24 h and their supernatant (SN Eos) was stored in aliquots at −80 °C. The purity of isolated primary human eosinophils was evaluated by flow cytometry immunophenotyping (see M&M paragraph below).

### Culture of MPM cells in 2D and in spheroids

The epithelioid M14K (RRID:CVCL_8102) and biphasic ZL34 (RRID:CVCL_5906) human MPM cell lines were cultured in 2D at 37 °C in DMEM medium (VWR, L0104-500) containing 2 mM l-glutamine, supplemented with 10% heat-inactivated FBS and 1% penicillin and streptomycin (10,000 U/ml) (*i.e.,* complete DMEM). Low-passage primary cell cultures were established from frozen MPM tumours samples and maintained in complete RPMI-1640 as previously described.^36^^,^^37^ Cell lines and primary cultures were regularly tested for mycoplasma contamination. M14K and ZL34 spheroids were generated using the liquid overlay method according to the protocol of Friedrich et al.^38^ Briefly, 96-well plates were coated with 50 μL agarose (Sigma) dissolved in DMEM (1.5% w/v) to render plates non-adhesive. M14K and ZL34 cells were added to the wells at a density of 1.5 × 10^4^/well and cultured for 72 h in presence of differentiated EOL1 supernatant (25% v/v SN Dif-EOL1). Spheroid growth and response to cisplatin (10 μM; Sigma–Aldrich Cat# C2210000) and pemetrexed (10 μM; Sigma–Aldrich Cat# Y0001539) were recorded daily with a CKX41 inverted microscope (Olympus). After transfer into a 24-well plate, spheroid adherence and cell migration (surface occupied by cells in mm^2^) were recorded with the CKX41 inverted microscope and quantified with the ImageJ software (RRID:SCR_00370).

### Flow cytometry immunophenotyping

After 2 washes with PBS-FBS 2%, Dif-EOL1 cells were labelled for 1 h on ice with 1 μg/mL of anti-IL-5Rα antibody (Invitrogen Cat# PA525159, RRID:AB_2542659). After 2 washes, cells were incubated for 45 min with 2 μg/mL AlexaFluor488 anti-rabbit IgG (Invitrogen Cat# A11008, RRID:AB_143165) conjugate as well as with 1 μg/mL anti-CD193 (CCR3) antibody coupled with allophycocyanin (APC) (eBioscience Cat# 15518046, RRID:AB_10853007).

CCR3^+^ primary human eosinophils were fixed in the dark in PBS containing 4% paraformaldehyde (PFA, Sigma Cat# 1004960700) for 10 min at room temperature. After 2 washes in PBS-FBS 2%, cells were labelled for 1 h at room temperature with 1 μg/mL anti-IL-5Rα and anti-Siglec-8 (Miltenyi Biotec Cat# 130-108-015, RRID:AB_2653436) antibodies. After 2 washes, cells were incubated for 45 min with 2 μg/mL AlexaFluor488 anti-rabbit IgG and AlexaFluor647 anti-mouse IgG1 (Invitrogen Cat# A21240, RRID:AB_2535809) conjugates.

For CLC-P/Gal10 detection, primary eosinophils and Dif-EOL1 cells were fixed in PBS containing 4% PFA and 0.5% Tween 20 overnight at 4 °C. After 2 washes with PBS containing 3% FBS and 0.5% Tween 20, cells were labelled for 1 h at room temperature with 10 μg/mL anti-CLC-P/Gal10 (Diaclone Cat# 852.960.000, RRID:AB_596462). After 2 washes, cells were incubated for 45 min with 2 μg/mL AlexaFluor488 goat anti-mouse IgG1 (Invitrogen Cat# A21121, RRID:AB_2535764) conjugate.

The labelled cells were recorded by flow cytometry (Cytoflex, Beckman Coulter, RRID:SCR_026067) and analysed with the Cytexpert software (Beckman Coulter, RRID:SCR_017217).

### Confocal microscopy

EOL1 progenitors and Dif-EOL1 cells were washed in PBS and fixed with 4% PFA for 10 min at room temperature in the dark. After 2 washes, PFA quenching with 50 mM glycine and permeabilization with PBS containing 0.5% Triton X-100 for 10 min, samples were incubated with 0.25 μg/mL APC-coupled anti-CCR3 (eBioscience Cat# 15518046, RRID:AB_10853007) and ActiGreen 488 ReadyProbes (ThermoFisher Cat# R37110) for 30 min at room temperature before mounting with Fluoroshield-DAPI (Sigma–Aldrich Cat# F6057).

Alternatively, primary eosinophils and Dif-EOL1 cells were washed in PBS and fixed with 4% PFA for 10 min at room temperature in the dark. After 2 washes, PFA quenching with 50 mM glycine and permeabilization with PBS containing 0.5% Triton X-100 for 10 min, samples were incubated overnight at 4 °C with 15 μg/mL mouse IgG1 anti-CLC-P/Gal10 monoclonal antibody (Diaclone Cat# 852.960.000, RRID:AB_596462) and 1 μg/mL rabbit antiserum specific for the major basic protein (MBP) (Invitrogen Cat# PA5102628, RRID:AB_2852025). After 2 washes with PBS, cells were labelled with 2 μg/mL AlexaFluor488 goat anti-mouse IgG1 (Invitrogen Cat# A21121, RRID:AB_2535764) and TexasRed goat anti-Rabbit IgG (Invitrogen Cat# T-6391, RRID:AB_2556779) conjugates for 30 min at room temperature before mounting with Fluoroshield-DAPI. The same protocol was used to reveal CLC-P/Gal10 in M14K cells except that membranes were stained with 5 μg/mL CellMask (Invitrogen Cat# C10045) before fixation with 4% PFA.

M14K and ZL34 cells were labelled with 10 μM carboxyfluorescein succinimidyl ester (CFSE, Abcam) for 7 min at 37 °C and washed in complete DMEM. CFSE-labelled M14K cells were co-cultured with primary eosinophils on coverslips for 24 h at a 1:1 ratio and fixed with 4% PFA for 10 min at room temperature in the dark. After permeabilization with 1% Triton X-100 in PBS for 10 min, cells were incubated in PBS containing 10% FBS for 30 min. After 1 wash with PBS, cells were labelled with 1 μg/mL APC-coupled anti-human tetraspanin (CD63) conjugate (Immunotools Cat# 21270636, Clone MEM-259) for 1 h at room temperature. Cells were stained with 5 μM DAPI (BioLegend Cat# 422801) and coverslips were mounted with Fluoroshield (Sigma–Aldrich Cat# F6182).

Paraffin-embedded biopsies from MPM patients were obtained from the institutional biobank of the CHU (Liege) after approval by the local Ethical Committee (case number 2020/45 and 2021/292). Biopsy sections were cut at 4 μm thickness and mounted on microscope slides, dewaxed with xylene and rehydrated in graded ethanol baths. Slices were boiled in citrate buffer at 120 °C for 20 min in a pressure cooker. Slides were permeabilized with PBS-0.5% Triton X-100 for 5 min and pre-incubated with PBS-10% FBS for 1 h at room temperature. Tumour sections were labelled with 0.25 μg/mL APC-coupled anti-CCR3 (eBioscience Cat# 15518046, RRID:AB_10853007) and 5 μg/mL mouse IgG1 anti-CLC-P/Gal10 monoclonal antibody (Diaclone Cat# 852.960.000, RRID:AB_596462) for 1 h at room temperature. Samples were then incubated with 2 μg/mL AlexaFluor488 goat anti-mouse IgG1 conjugate (Invitrogen Cat# A21121, RRID:AB_2535764) and DAPI for 1 h at room temperature.

All confocal images were obtained with either Zeiss LSM 880 AiryScan Elyra S1 (RRID:SCR_020925) or Zeiss LSM 980 AiryScan Elyra S2 (RRID:SCR_025048) confocal microscopes equipped with x40 and x63–1.4 oil immersion objectives and analysed with Imaris software (Zeiss, RRID:SCR_007370).

Validation of all the antibodies and cell lines are available on the manufacturer's website and can be found in Reagent Validation File.

### Time-lapse imaging

After differentiation, Dif-EOL1 cells (1 × 10^6^ cells) were labelled with 10 μM CFSE for 7 min at 37 °C and washed in complete RPMI. CFSE-labelled Dif-EOL1 cells (1 × 10^5^ cells/well) were then co-cultured with M14K or ZL34 (ratio 1:1) in a 96-well plate at 37 °C. Cell cultures were monitored hourly by Incucyte imaging S3 Live-Cell system equipped with a 20X objective (Sartorius, RRID:SCR_023147).

### Analysis of apoptosis by flow cytometry

M14K and ZL34 cells were cultured for 48 h in 2D monolayers in a 24-well plate (2.5 × 10^4^ cells/well) in the presence of 25% eosinophil supernatant (SN Dif-EOL1 or SN Eos) or N-terminal His-tag recombinant human CLC-P/Gal10 (1 and 5 μg/ml; Novus Biological Cat# NBP1-51096). Experimental conditions also included antibody-mediated depletion of CLC-P/Gal10. For this purpose, SN Dif-EOL1 and SN Eos were incubated with 1 μg/mL anti-CLC/Gal10 antibody (Diaclone Cat# 852.960.000, RRID:AB_596462) for 1 h at room temperature. After addition of anti-mouse IgG magnetic microbeads (Miltenyi Biotec, Cat# 130-048-402, RRID:AB_244361) for 30 min, CLC-P/Gal10 was depleted from SN Dif-EOL1 and SN Eos by magnetic sorting.

Forty-eight hours after culture with SN Dif-EOL1, SN Eos or CLC-P/Gal10-depleted medium, MPM cells were treated with cisplatin (10 μM; Sigma–Aldrich, Cat# C2210000) and pemetrexed (10 μM; Sigma–Aldrich, Cat# Y0001539) for 48 h before further analyses without drug washout. Apoptosis was evaluated by phosphatidylserine exposure and plasma membrane integrity using Annexin V-FITC (Immunotool, Cat# 31490013) and propidium iodide (PI, BioLegend Cat# 421301) as described by the manufacturer. Ten thousand events were recorded by flow cytometry (FacsCanto II, BD Bioscience, RRID:SCR_018056) and analysed with the FlowJo software (version X.0.7, BD Bioscience, RRID:SCR_008520). Annexin V^+^ PI^−^ and Annexin V^+^ PI^+^ cells were considered as undergoing early and late apoptosis, respectively.

DNA fragmentation consecutive to apoptosis was quantified by cell cycle analysis upon ethanol fixation and PI staining. After overnight permeabilization with ethanol 70% (v/v) at −20 °C, MPM cells were digested with 100 μL RNAse A (50 μg/mL; Sigma–Aldrich, Cat# 10109169001) in PBS containing 0.1% Tween-20 for 30 min at 37 °C and stained with PI (20 μg/mL) for 10 min in the dark at room temperature. Ten thousand events were recorded by flow cytometry (Cytoflex, Beckman Coulter, RRID:SCR_026067) and, after exclusion cell doublets, analysed with the CytExpert software (version 2.4, Beckman Coulter, RRID:SCR_017217).

### Senescence analysis

M14K cells were cultured for 48 h in 2D monolayers in a 24-well plate (2.5 × 10^4^ cells/well) in the presence of 25% eosinophil supernatant (SN Dif-EOL1 or SN Eos) or N-terminal His-tagged recombinant human CLC-P/Gal10 protein (Novus Biological Cat# NBP1-51096) at 0.1 μg/ml. Cells were then treated with 10 μM cisplatin (Sigma–Aldrich, Cat# C2210000) and 10 μM pemetrexed (Sigma–Aldrich, Cat# Y0001539) for 48 h before staining the senescence-associated (SA-)β-galactosidase according to manufacturer's instructions (CellSignaling Cat# 9860). Briefly, cells were washed, fixed for 15 min at room temperature and stained overnight at 37 °C for SA-β-galactosidase in a sealed plate. Cells labelled for SA-β-galactosidase were counted in ten different fields with a CKX41 inverted microscope (Olympus, RRID:SCR_023725), and the proportion of positive cells was calculated using ImageJ (RRID:SCR_00370).

### Titration of CLC-P/Gal10 in eosinophil supernatant, patients’ sera and pleural effusions

Diagnoses were established by both fluid cytology and immunohistochemical staining of pleural biopsies performed by the pathology department at Laennec Hospital (St-Herblain) and externally confirmed by Mesopath, the French panel of pathology experts to avoid diagnosis heterogeneity.^39^ Definite diagnosis was based on the Mesophath decision. Depending on their condition, patients with mesothelioma were treated with platinum-based chemotherapy or received palliative medical care. Recruited patients had received no prior anticancer therapy and gave signed informed consent. After approval by local ethical committee (CPP Ouest-IV-Nantes), sera (n = 39), pleural effusions from all patients with mesothelioma (n = 81) and controls samples (n = 20), regardless of sex, were collected before the study as described by Gueugnon et al.^39^ and in accordance with the standards established by the Declaration of Helsinki. Collected samples and the associated clinical information were registered in a database (DC-2017-2987) validated by the French ministry of research. CLC-P/Gal10 titrations were performed on all collected samples from the cohort with the Human Galectin-10 ELISA Kit (Invitrogen Cat# EH204RB) according to manufacturer instructions. Pleural effusions, sera and culture supernatants were diluted 10-fold and added to the ELISA plate. Optical densities were recorded at λ = 450 nm with the Multiskan FC Microplate Photometer (ThermoFisher).

### RNA sequencing and bioinformatics

RNA was extracted from M14K and ZL34 cells using the NucleoSpin RNA Plus kit as described by the manufacturer (Macherey–Nagel Cat# 740984.50) and quantified with a Nanodrop 2000 spectrophotometer. Sequencing of the libraries (2 x 150 bp) with a depth of 30M paired reads per sample was performed by Macrogen Europe on a NovaSeq 6000 system (Illumina, San Diego, CA, USA).

Quality controls of FASTQ reads included base quality, sequence duplications and adapter contents (FASTQ tools version 0.11.9). Illumina universal adapters as well as low quality and short reads were filtered out with Trimmomatic (version 0.39). Reads corresponding to rRNA were removed with bwa mem (version 0.7.17). rRNA-free and trimmed reads were mapped to the human genome (hg18, Genome Reference Consortium GRCh38) using STAR (version 2.7.9.a). Aligned reads were further marked for duplicates with MarkDuplicates (Picard version 2.26.3). The read count table was eventually generated using Featurecounts (Rsubread version 2.0.1).

Differential expression analysis between experimental conditions was performed using the R/Bioconductor DESeq2 package (version 1.34.0). Differentially expressed genes (DEGs) (p-adj. ≤ 0.05 and |Log_2_FC| > 1) were obtained in each comparison and ranked according to adjusted p-values (p-adj.) correcting for multiple comparisons. Functional pathway analysis was performed by over-representation (ORA) and gene set enrichment analysis (GSEA). ORA analysis of significant genes (p-adj. ≤ 0.05) was performed against Gene Ontology (GO) for molecular function (MF), Biological Process (BP) and Kyoto Encyclopedia for Genes and Genomes (KEGG) using g:OSt from the gprofiler2 package (version 0.2.1).^40^ GSEA of DEGs was performed against GO: BP using gseGO from the clusterProfiler package (version 4.2.2).^41^

Publicly available datasets analysed in this study can be found on the library: https://www.ncbi.nlm.nih.gov/bioproject/PRJNA1049163, accessed on December 12, 2023.

### Mouse model

All procedures were approved by the Ethical Review Board (protocol #2366) and performed according to the Federation of Laboratory Animal Science Association (FELASA) guidelines. Mice were housed in conventional ventilated cages in accordance with federal guidelines. Investigators were blinded to group allocation during experiments. Groups were allocated randomly following tumour size to obtain the minimal number of mice per group. Cofounders were not controlled. Sample-size based on tumour growth was calculated *a priori* with G∗Power 3.1.9.6 (RRID:SCR_013726) by estimating the Cohen size effect index based on previous experiments (*i.e.,* large). Using an effect size of 2.5, risk α = 0.05 and with an estimated power of 0.971, the target sample size of 5 mice per group was reached.

Fifty-four six-week-old C57BL/6 mice (Janvier Labs, RRID:IMSR_RJ:C57BL-6JRJ) were inoculated subcutaneously in both flanks with 1.5 × 10^6^ syngeneic AK7 epithelioid mesothelioma cells. When the tumour reached ∼150 mm^3^, eosinophilia was induced by daily intraperitoneal (i.p.) injections of recombinant mouse IL-5 (Immunotools Cat #12340053) and IL-33 (Immunotools Cat #12340335) at 5 and 20 ng/g of body weight (gbw), respectively. Upon regular blood sampling from the tail vein, eosinophil counts were measured with an hematocytometer (Advia 2120i; Siemens Healthineers). After 5 days, eosinophilia was maintained with i.p. injections of either IL-5+IL-33 or IL-5 alone. When the tumour volume reached ∼500 mm^3^, mice were treated i.p. with PBS (mock) or with 6 μg/gbw cisplatin (Sigma–Aldrich, Cat# 232120-50) and 150 μg/gbw pemetrexed (Sigma–Aldrich, Cat# Y0001539). Two days before this chemotherapy, eosinophils were depleted with 15 μg of anti-Siglec-F monoclonal antibody given i.p (Bio-Techne Cat# MAB17061, RRID:AB_2286029). Control group (mock) included mice that were inoculated subcutaneously with AK7 but did not receive any treatment. Tumour dimensions (L = length, W = width, H = height) were measured tri-weekly and volumes were calculated using the hemi-ellipsoid formula V = LxWxHxπ/6.

### Ethics

The use of human buffy coats from healthy donors (Red Cross of Belgium) was approved by the institutional ethic committee of the University Hospital (CHU, Sart-Tilman) under the reference #2012/8. The use of biopsies from the institutional biobank of the CHU (Liege) was approved by the local Ethical Committee (studies number 2020/45 and 2021/292). Patients recruited for the collection of pleural fluids gave their signed informed consent. After approval by the local ethical committee (CPP Ouest-IV-Nantes), pleural fluids were collected in accordance with the standards established by the Declaration of Helsinki. Animal experimentation was approved by the institutional ethical review board for animal use (protocol #2366), performed according to FELASA guidelines and following ARRIVE guidelines.

### Statistics

Statistical analyses were performed using GraphPad Prism 10.1.1 (RRID:SCR_002798) for *in vitro* experiments or R (v4.1.1) and RStudio 2022.07.1 + 554 (RRID:SCR_000432) for mice experiments. The Shapiro–Wilk test was used to verify if continuous variables followed a normal distribution. For comparisons between 2 populations, in case of normal distribution, the variance of the means was compared by t-test. If populations were not following a normal distribution, the variance of the means was compared with a Welch's test.

For comparisons between more than 2 populations, the homogeneity of the group variances was evaluated with the Brown–Forsythe test. When populations followed normal distributions and had similar variances, the variance of the means was compared by one-way ANOVA followed by Tukey's multiple comparison test. If the variances were significantly different, the variance of the means was compared by one-way ANOVA followed by Brown–Forsythe and Welch. Finally, if populations were not following a normal distribution, the variance of the means was compared with the nonparametric one-way ANOVA followed by Dunnett's T3 multiple comparison test.

For mice experiments, growth indexes were calculated using the formula ((V_F_ – V_C + P_)/V_C + P_) where V_F_ is the tumour volume at the end of the experiment and V_C + P_ is the tumour volume at the time of cisplatin and pemetrexed injection. Based on these growth indexes, two-way ANOVA with categorical variables “eosinophilia” and “chemotherapy” for the first experiment, or “eosinophilia” and “anti-Siglec-F” for the second experiment was performed. After checking if the populations were following a normal distribution with Shapiro–Wilk and evaluating the variance homogeneity with Levene's test, the variance of the means was compared with two-way ANOVA. Finally, the actual effect size (f^2^) was determined after the experiment by calculating the partial η^2^ for the interaction between “eosinophilia” and “chemotherapy” and for the interaction between “eosinophilia” and “anti-Siglec-F”. The power of the analysis was calculated using G∗Power 3.1.9.6 (RRID:SCR_013726). No animals were excluded from the analysis.

Preliminary exploration of patient data survey revealed a potential link between Gal10 and survival duration. Two groups of patients were created based on their CLC-P/Gal10 level in PEs: Low Gal10 ≤ 123.8 ng/mL of PE *vs.* High Gal10 > 123.8 ng/mL of PE. This threshold was chosen based on the maximization of between sum of squares of survival times between resulting groups. An Aalen's additive regression model was conducted to assess the consistency of the effects of Gal10 groups on survival over time. As the Aalen's model showed a discontinuity in the effects after about one year of survival, a landmark approach was chosen to analyse the effect of Gal10 on survival, with a milestone at the first year of survival. Two Kaplan–Meier non-parametric models were then fitted on patient's survival data, respectively on first year survival and after the first year of survival. Analyses were performed using R (v4.2.1) and packages survival 3.4-0 and survminer 0.4.9.

### Role of funders

The funders did not have any role in the study design, data collection, data analyses, interpretation, or writing of this article.

## Results

### The culture supernatant from differentiated EOL1 impacts mesothelioma cell response to cisplatin and pemetrexed chemotherapy

**Fig. 1:**
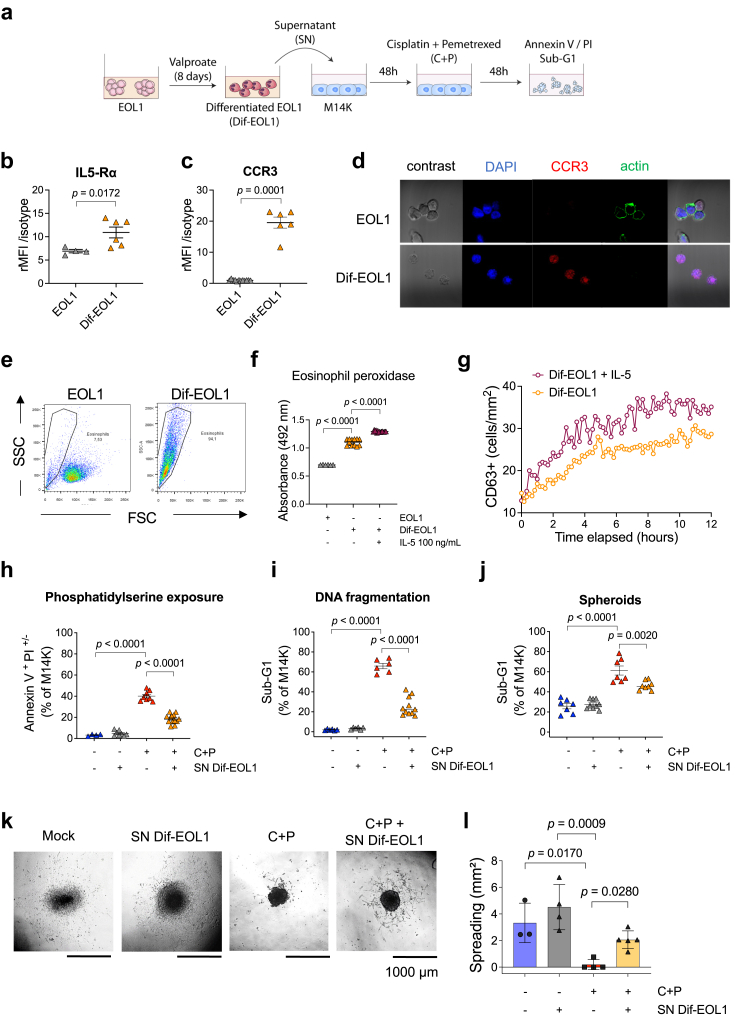
**Eosinophils differentiated from EOL1 cells inhibit the response to cisplatin and pemetrexed**. **(a)** Valproate-differentiated EOL1 (Dif-EOL1) supernatant (SN) was added to M14K mesothelioma cells at 25% v/v for 48 h. M14K were then treated with 10 μM cisplatin and 10 μM pemetrexed (C + P) for 48 h. After fluorescent labelling of IL-5Rα and CCR3, EOL1 progenitors and Dif-EOL1 were analysed by flow cytometry. The relative Median Fluorescence Intensity (rMFI) corresponds to the ratio of fluorescence intensities associated with IL-5Rα **(b)** and CCR3 **(c)** with control isotypes. Bars represent mean ± standard deviation (SD) from 6 independent experiments. **(d)** Dif-EOL1 were labelled for CCR3 and actin, stained with DAPI and analysed by confocal microscopy (magnification 40×). **(e)** Flow cytometry was used to discriminate EOL1 progenitors from Dif-EOL1 based on size (forward scatter; FSC) and granulometry (side scatter; SSC). **(f)** Eosinophil peroxidase activity in EOL1 and Dif-EOL1 cells (stimulated with mock or IL-5; 100 ng/mL). Absorbance of the chromogenic substrate (OPD) was measured at 492 nm with a spectrophotometer. **(g)** CD63-positive cells (number/mm^2^) were recorded by time-lapse microscopy (Incucyte imaging S3 Live-Cell system equipped with a 20X objective) in Dif-EOL1 cultures in presence or not of IL-5 (100 ng/mL). **(h)** Percentages of apoptotic M14K cells (2D) were determined by flow cytometry after Annexin V/propidium iodide (PI) staining. Early (Annexin V^+^ PI^−^) and late (Annexin V^+^ PI^+^) apoptotic cells were counted for each condition. **(i)** After ethanol permeabilization and PI staining, the cell cycle profiles (2D) were analysed by flow cytometry. The percentages of cells with fragmented genomic DNA (*i.e.,* Sub-G1) were evaluated. **(j)** M14K spheroids were generated by the liquid overlay method for 72 h in presence or absence of Dif-EOL1 supernatant. After treatment with C + P for 48 h, the proportion of M14K cells in Sub-G1 was determined by flow cytometry after spheroid dissociation, cell permeabilization and PI staining. **(k)** After transfer of the spheroids into an adherent 24-well plate, cell migration was monitored with an Olympus CKX41 microscope. **(l)** The surface occupied by the cell culture in mm^2^ was measured after 24 h. Data are expressed as means ± SD, each point representing an independent test. Normality was checked by Shapiro–Wilk and equality of the variances was checked by Brown–Forsythe. Variance of the means was compared by t-test (panels b and c) or by one-way ANOVA followed by Tukey's multiple comparison test (panels f, h, i, j and l).

An experimental model *in cellulo* was designed to evaluate the effect of eosinophils on mesothelioma response to chemotherapy. EOL1 eosinophilic cells were differentiated (Dif-EOL1) for 8 days with valproate, a histone deacetylase (HDAC) inhibitor (Fig. 1a). An optimal concentration of 2 mM valproate promoted the expression of two characteristic eosinophil markers: IL5-Rα. Fig. 1b) and CCR3 (Fig. 1c). Upon differentiation, the actin cytoskeleton underwent depolymerization as revealed by lack of phalloidin labelling (Fig. 1d). Dif-EOL1 cells were more granular and smaller than EOL1, as indicated by the side (SSC) and forward (FSC) scatters (Fig. 1e). Consistent with their phenotype, Dif-EOL1 also demonstrated higher levels of eosinophil peroxidase activity (Fig. 1f). Moreover, live cell imaging demonstrated that IL-5 increased the expression of CD63 thereby indicating eosinophil activation and functional degranulation (Fig. 1g). These results confirmed that Dif-EOL1 cells were phenotypically representative of functional human eosinophils.

To study the impact of eosinophils on chemotherapy, M14K mesothelioma cells were cultured with 25% v/v of Dif-EOL1 cell supernatant (SN Dif-EOL1) for 48 h and treated with 10 μM cisplatin and 10 μM pemetrexed (C + P) for 48 additional hours (Fig. 1a). Flow cytometry revealed that the percentages of Annexin V^+^ PI^+/−^ cells increased in presence of the C + P regimen indicating, as expected, the onset of apoptosis (Fig. 1h). Preincubation of M14K cells with SN Dif-EOL1 significantly attenuated the proapoptotic effect of the C + P regimen compared to the control medium (*p* < 0.0001). As another hallmark of apoptosis, DNA fragmentation reflected by the proportion of Sub-G1 cells was reduced in presence of SN Dif-EOL1 (Fig. 1i). To extend these conclusions obtained in 2D cultures, the effect of the Dif-EOL1 supernatant was evaluated in spheroids. In this 3D model, the SN Dif-EOL1 significantly decreased apoptosis induced by the C + P regimen (Fig. 1j). Upon transfer into a C + P free medium, adherence and outgrowth of the spheroids precultured with SN Dif-EOL1 was slightly improved compared to the mock control although the effect was not statistically significant (Fig. 1k–l). The Dif-EOL1-conditioned medium ameliorated spreading of C + P treated spheroids (*p* = 0.0280, Fig. 1l).

These experiments thus demonstrate that the supernatant of Dif-EOL1 attenuated the pro-apoptotic effect of C + P in epithelioid mesothelioma M14K cells. In another cell model involving non-epithelioid mesothelioma (ZL34), the proportion of Annexin V^+^/PI^+/−^ events induced by C + P regimen was also significantly reduced by SN Dif-EOL1 (Supplementary Fig. S1). In the experimental settings, the C + P regimen mainly induced late apoptosis (*i.e.*, Annexin V^+^/PI^+^; Supplementary Fig. S2). DNA fragmentation analysis revealed that ZL34 cells most frequently arrested in S phase in presence of C + P (Supplementary Fig. S3). This S phase blockade induced by C + P was significantly reduced in presence of SN Dif-EOL1.

Collectively, these data revealed that the culture supernatant from differentiated EOL1 impacted mesothelioma cell response to C + P chemotherapy.

### Eosinophil-associated factors affect binding functions of the mesothelioma transcriptome

To characterise the mechanisms promoted by the eosinophil-conditioned medium, the kinetics of the experimental protocol was modified. When the SN Dif-EOL1 and the C + P regimen were added concomitantly to the M14K or ZL34 mesothelioma cells, the anti-apoptotic effect of the eosinophilic supernatant was lost, as reflected by the percentage of Annexin V^+^ PI^+/−^ cells (Supplementary Fig. S4a-b). In the spheroid model, DNA fragmentation in M14K and S phase arrest in ZL34 were also unaffected by SN Dif-EOL1 (Supplementary Fig. S4d-e, respectively). This result indicated that preincubation of mesothelioma cells with the SN Dif-EOL1 was required to attenuate the effect of C + P. This delay suggested that the eosinophilic supernatant promoted molecular changes in mesothelioma cells.

**Fig. 2:**
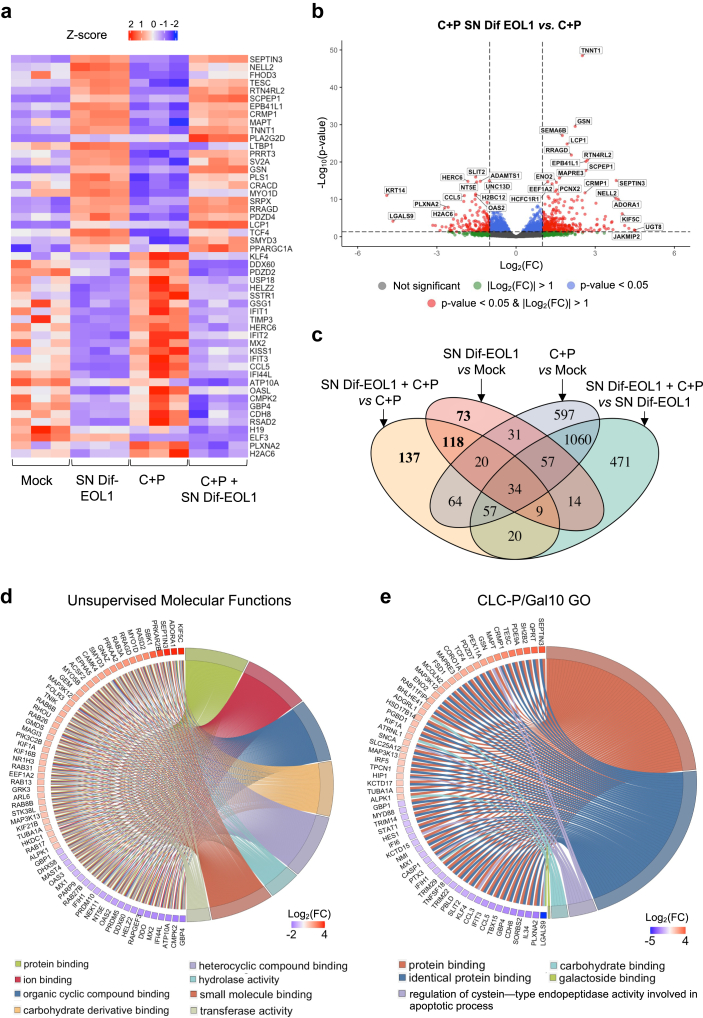
**Conditioned media of differentiated EOL1 cultures induce transcriptomic changes in M14K cells**. **(a)** Unsupervised heatmap of the 25 most significant up-regulated (red) and down-regulated (blue) genes in the transcriptome of M14K cells. Experimental data with the control (Mock), Dif-EOL1 supernatant (SN) and/or cisplatin + pemetrexed (C + P) were deduced from 3 independent replicates. **(b)** Volcano plot of differentially expressed genes (DEGs) in conditions C + P and SN Dif-EOL1 *vs.* C + P. Genes with |Log_2_(FC)| > 1 and -Log_10_pvalue >1.3 (p-adj. threshold: 0.05) are marked in red. **(c)** Venn diagram of significant DEGs in the different conditions (Mock, C + P, Dif-EOL1 SN, C + P and Dif-EOL1). The numbers of genes impacted by SN Dif-EOL1 are in bold. **(d)** Representative chord diagram of the most significant pathways affected in Gene Ontology Molecular Functions (GO:MF) in conditions C + P and SN Dif-EOL1 *vs.* C + P. Pathways (right side) are linked to the genes (left side) according to their Log_2_(FC). The names of the pathways are provided below the diagram. **(e)** Representative chord diagram of the most significant pathways associated with CLC-P/Gal10 in GO:MF.

To get deeper insight into the mechanisms promoted by the eosinophil-conditioned medium, the transcriptome of M14K and ZL34 cells cultured in presence of the SN Dif-EOL1 and/or C + P regimen was evaluated by RNA sequencing. The heatmap of differentially expressed genes (DEGs) indicated that the SN Dif-EOL1 and/or C + P regimen significantly modified transcription in the two mesothelioma cell lines (Fig. 2a, Supplementary Fig. S5). In particular, the volcano plot highlighted specific changes (DEGs at p-adj. < 0.05 and |Log_2_FC| > 1) that correlated with the effect of the Dif-EOL1-conditioned medium upon C + P treatment of M14K cells (Fig. 2b). Among these, 137 unique DEGs were associated with SN Dif-EOL1 + C + P *vs* C + P as illustrated by the Venn diagram (Fig. 2c).

To obtain a comprehensive view of the underlying mechanisms, an unsupervised analysis of the transcriptomic data set was performed. Gene Ontology (GO) comparison and ORA pathway enrichment analysis revealed that protein binding (GO:0005515, p-adj. = 9.59 × 10^−150^), ion binding (GO:0043167, p-adj. = 1.63 × 10^−44^), organic cyclic compound binding (GO:0097159, p-adj. = 2.09 × 10^−22^), heterocyclic compound binding (GO:1901363, p-adj. = 2.19 × 10^−21^), hydrolase activity (GO:0016787, p-adj. = 2.71 × 10^−20^), small molecule binding (GO:0036094, p-adj. = 5.68 × 10^−19^) and transferase activity (GO:0016740, p-adj. = 4.36 × 10^−16^) characterised the impact of the SN Dif-EOL1 on the C + P response (Fig. 2d). This analysis suggested that a soluble factor secreted in the SN Dif-EOL1 primarily affected the binding functions in mesothelioma cells. In particular, pathways associated with the Charcot-Leyden Crystal protein/galectin-10 (CLC-P/Gal10), a protein predominant in human eosinophils, were included in the top list of the GO analysis. Indeed, a series of molecular functions of the CLC-P/Gal10 protein (*i.e.*, GO:0005515, GO:0042802, GO:0043281, GO:0030246 and GO:0016936) closely characterised the effect of the SN Dif-EOL1 on the C + P response (Fig. 2e).

The Gene Set Enrichment analysis (GSEA) highlighted a series of GOs involved in C + P response of M14K (Supplementary Fig. S6) and ZL34 (Supplementary Fig. S7) cells. Supplementary Figs. S8–S9 showed that GOs involved in chemotherapy response were suppressed by the Dif-EOL1 supernatant. However, the unsupervised GSEA analysis of GOs also revealed several unexpected pathways that illustrated the complexity of the mechanisms involved (Supplementary Tables S1–S4).

These transcriptomic analyses thus indicated that the SN Dif-EOL1 significantly affected the binding functions in mesothelioma cells, possibly via soluble mediators.

### Dif-EOL1-derived CLC-P/Gal10 affects MPM cells response to C + P chemotherapy

**Fig. 3:**
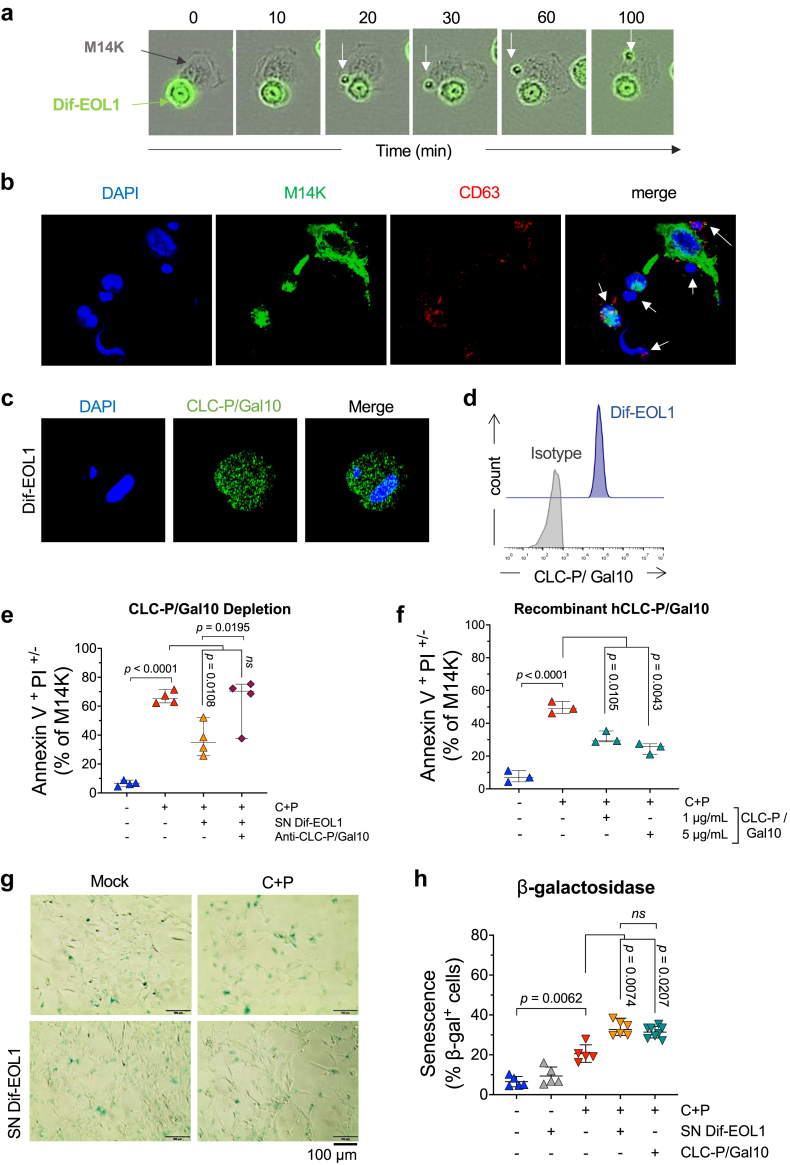
**CLC-P/Gal10 inhibits MPM cell response to chemotherapy**. **(a)** Incucyte time-lapse imaging of M14K cells (black) and CFSE-stained Dif-EOL1 cells (green). The white arrow shows a CFSE-labelled granule interacting with M14K cells. **(b)** Confocal microscopy of primary human eosinophils (Eos) co-cultured with CFSE-labelled M14K cells. After 24 h, cells were fixed, permeabilized, stained with DAPI and labelled with an anti-CD63 APC conjugate (in red). Images were acquired using a Zeiss LSM 880 AiryScan Elyra confocal microscope equipped with a x63–1.4 oil immersion objective. White arrows indicate eosinophils. **(c)** Confocal analysis of Dif-EOL1 cells labelled with DAPI (blue), with an anti-CLC-P/Gal10 antibody and with an AlexaFluor488 conjugate (green). Images were acquired using a Zeiss 880 or 980 Airyscan Elyra confocal microscope equipped with a x63–1.4 oil immersion objective. **(d)** Representative histogram plot of CLC-P/Gal10 expression acquired by flow cytometry. Dif-EOL1 cells were labelled as described in panel c. **(e)** Apoptosis of M14K cells in presence of SN Dif-EOL1, SN Dif-EOL1 depleted in CLC-P/Gal10 and/or C + P. CLC-P/Gal10 was depleted from the supernatant by antibody-mediated depletion. Then M14K cells were cultured for 48 h in presence of SN Dif-EOL1 depleted in CLC-P/Gal10. After treatment with C + P for 48 h, cells were labelled with Annexin V/PI and analysed by flow cytometry. **(f)** Recombinant human CLC-P/Gal10 (0.1, 0.5, 1 and 5 μg/mL) was added in M14K culture medium for 48 h before treatment with C + P. Apoptosis of M14K cells was determined by flow cytometry after Annexin V/PI labelling. **(g)** Representative images of senescent M14K cells in presence of conditioned medium of differentiated EOL1 (SN Dif-EOL1). M14K cells were cultured for 48 h in presence of mock, SN Dif-EOL1 (25% v/v) or recombinant human CLC-P/GAL10 (1 μg/mL). M14K cells were then treated with 10 μM cisplatin and 10 μM pemetrexed for an additional 2 days. Senescence-associated β-galactosidase (SA-β-gal) activity at pH 6.0 was visualized with an Olympus CKX41 inverted microscope equipped with a 20X objective. **(h)** The percentages of SA-β-gal positive cells were counted in ten different fields. Data are expressed as median ±95% CI, each dot representing an independent test. Normality was checked by Shapiro–Wilk and equality of the variances was verified by Brown–Forsythe and Welch. Variance of the means was compared by one-way ANOVA followed by either Tukey's (panels e and f) or Dunnett's T3 (panel h) multiple comparison test.

To further characterise the interplay between eosinophils and mesothelioma cells, cocultures of Dif-EOL1 and M14K cells were analysed by time-lapse imaging. To trace eosinophilic factors, the protein content of Dif-EOL1 cells was labelled with CFSE prior to the coculture. Fluorescence microscopy showed that Dif-EOL1 cells interacted with M14K cells (Fig. 3a). In addition, CFSE-stained components migrated from eosinophils to the M14K cells, suggesting secretion and transfer of eosinophilic protein factors. This interpretation was validated by confocal microscopy upon tetraspanin labelling (CD63 in red; Fig. 3b). Representing approximately 7–10% of eosinophil cytoplasmic proteins, CLC-P/Gal10 was reported to be a main component of the granules.^26^^,^^42^^,^^43^ Consistently, confocal microscopy and flow cytometry showed that Dif-EOL1 indeed expressed large amounts of CLC-P/Gal10, as expected (Fig. 3c and d, Supplementary Fig. S10). The C + P treatment and factors in MPM supernatant did not significantly affect CLC-P/Gal10 secretion by eosinophils (Supplementary Figs. S11 and S12).

To test whether resistance to C + P chemotherapy involved CLC-P/Gal10, the protein was directly removed from the Dif-EOL1-conditioned medium by antibody-mediated depletion. In the absence of CLC-P/Gal10, the Dif-EOL1 SN did not impair C + P-induced apoptosis of M14K cells (Fig. 3e). Conversely, the addition of a recombinant human CLC-P/Gal10 protein (1 and 5 μg/ml) reduced the proapoptotic activity of C + P regardless of the dose, thereby mimicking the effect of SN Dif-EOL1 (Fig. 3f).

To further evaluate the relevance of this observation, the response to CLC-P/Gal10 of low-passage cells isolated directly from tumours representative of the heterogeneity of MPM was analysed. Three primary cells representing the major histological subtypes were selected: MPM_07 (epithelioid), MPM_80 (sarcomatoid) and MPM_27 (biphasic). The susceptibility to C + P was different in the 3 cell cultures. Indeed, the concentrations of C + P inducing 50% apoptosis (IC50) in these primary cells ranged from 10 μM (MPM_27 and MPM_80) to 30 μM (MPM_07) (Supplementary Fig. S13). In presence of CLC-P/Gal10, the proportion of apoptotic cells with fragmented DNA (*i.e.,* Sub-G1) was affected at different levels in the 3 primary cells: dose-dependent decrease in MPM_07, reduction in MPM_80 up to 1 μg/mL then no effect at higher concentrations, and no response in MPM_27 (Supplementary Fig. S14). There was thus a spectrum of responses to CLC-P/Gal10 in different mesothelioma subtypes.

Since senescence is closely associated with chemotherapy resistance,^44^^,^^45^ M14K cells were cultured in presence of Dif-EOL1 SN and CLC-P/Gal10 prior to C + P treatment. Staining for SA-β-gal activity indicated that Dif-EOL1 SN and CLC-P/Gal10 significantly increased the proportion of senescent cells induced by chemotherapy (Fig. 3g and h).

These results demonstrated that CLC/Gal10 protein expressed by EOL1-derived eosinophils affected the response of MPM cells to C + P chemotherapy.

### CLC-P/Gal10 expressed by primary human eosinophils mediates chemoresistance

**Fig. 4:**
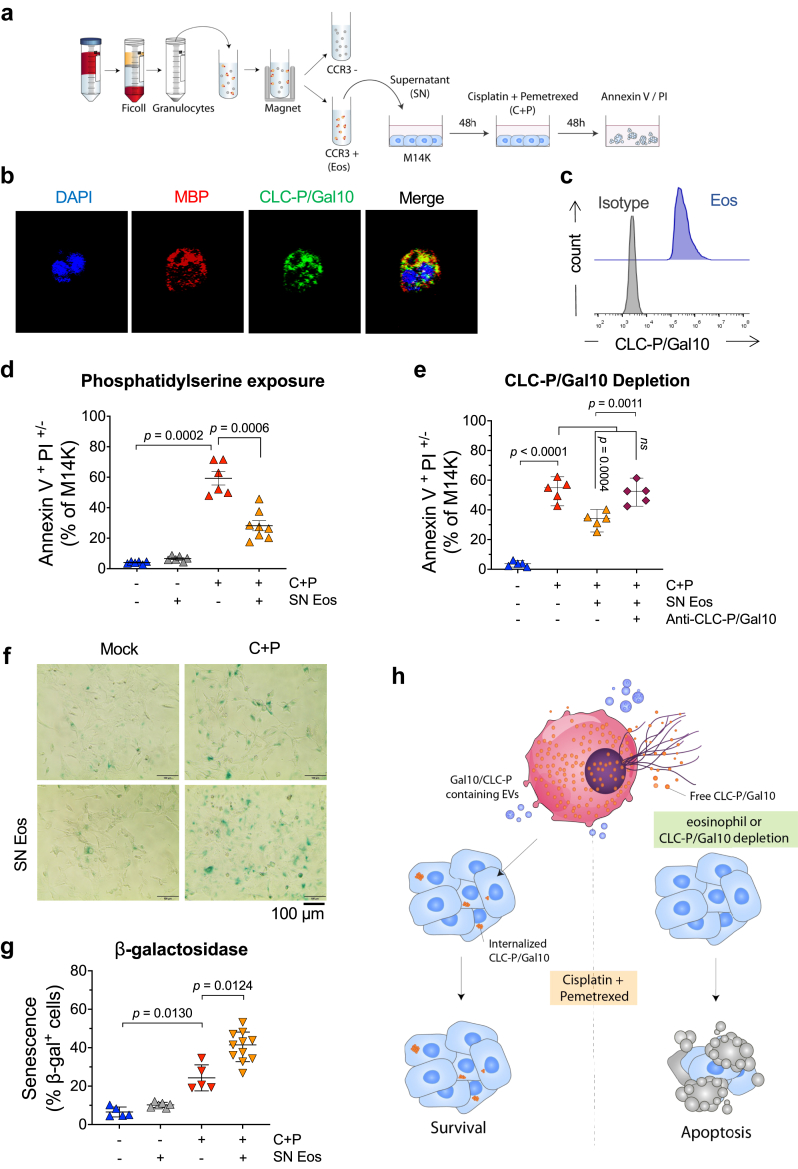
**CLC-P/Gal10 expressed by primary eosinophils impairs the cytotoxic activity of cisplatin and pemetrexed**. **(a)** Schematic representation of the experimental protocol for the isolation of primary human eosinophils. Primary eosinophils were purified from the polymorphonuclear cell (granulocytes)-rich fraction of peripheral blood by Ficoll gradient centrifugation and positively selected by magnetic-activated cell sorting using an anti-CCR3 antibody. The culture supernatant of CCR3-positive eosinophils (SN Eos) was collected after 24 h and added at a ratio of 25% (v/v) to M14K cells for 48 h. Then, M14K cells were treated with C + P for 48 h and analysed by flow cytometry after Annexin V/PI labelling. **(b)** Purified CCR3^+^ eosinophils were fixed, permeabilized and labelled with DAPI (blue), CLC-P/Gal10 (green) and MBP (red). Images were acquired using a Zeiss 980 Airyscan Elyra confocal microscope equipped with a x63–1.4 oil immersion objective. **(c)** Representative histogram plot of CLC-P/Gal10 expression acquired by flow cytometry. Primary eosinophils were labelled with an anti-CLC-P/Gal10 antibody and an AlexaFluor488 conjugate. **(d)** Apoptosis of M14K cells in presence of SN Eos and/or C + P. M14K cells were cultured with SN Eos for 48 h. After treatment with C + P for 48 h, cells were labelled with Annexin V/PI and analysed by flow cytometry. **(e)** Same as in panel d except that CLC-P/Gal10 protein was depleted from SN Eos by using an anti-Gal10 antibody. **(f)** Representative images of senescent M14K cells in presence of SN Eos. M14K cells were cultured for 48 h in presence of SN Eos treatment with 10 μM cisplatin and 10 μM pemetrexed for an additional 2 days. Senescence-associated β-galactosidase (SA-β-gal) activity at pH 6.0 was visualized with an Olympus CKX41 inverted microscope equipped with a 20X objective. **(g)** The percentages of SA-β-gal positive cells were counted in ten different fields. Data are expressed as median ±95% CI, each dot representing an independent test. Normality was checked by Shapiro–Wilk and equality of the variances were determined by Brown–Forsythe and Welch. Variance of the means was compared by one-way ANOVA followed by Tukey's (panel e) or Dunnett's T3 (Panels d and g) multiple comparison test. (**h**) Graphical summary of conclusions drawn from cell culture experiments.

The advantage of EOL1 progenitor-derived eosinophils was that, although not all cells underwent terminal differentiation, they all belonged to the same eosinophil-committed lineage. To extend the conclusions to primary cells, eosinophils (Eos) were isolated from peripheral blood of healthy donors using magnetic-activated cell sorting with an anti-CCR3 antibody (Fig. 4a). The purity of the isolated population was controlled by flow cytometry using Siglec-8 and IL-5Rα markers. Confocal microscopy confirmed that purified Eos expressing major basic protein (MBP) also stained positive for CLC-P/Gal10 (Fig. 4b and c).

To investigate the role of primary eosinophils in MPM chemoresistance, Eos-conditioned supernatant (SN Eos) was added to M14K cells 48 h prior to C + P treatment. The apoptotic response of M14K cells to C + P was significantly reduced in presence of the SN Eos. Compilation of 6 independent experiments with primary cells from different donors confirmed this conclusion statistically (*p* = 0.0006; Fig. 4d). Antibody-mediated depletion of CLC-P/Gal10 reverted the anti-apoptotic activity of the SN Eos in presence of C + P (*p* = 0.0011; Fig. 4e). The SN Eos significantly increased the SA-β-gal activity induced by chemotherapy (Fig. 4f). Compilation of 5 independent experiments with supernatants from different donors confirmed this conclusion statistically (*p* = 0.0124; Fig. 4g).

These experiments thus revealed that CLC-P/Gal10 produced by primary human eosinophils impaired the chemotherapeutic response of MPM cells (Fig. 4h).

### CLC-P/Gal10 is present in the tumour microenvironment

**Fig. 5:**
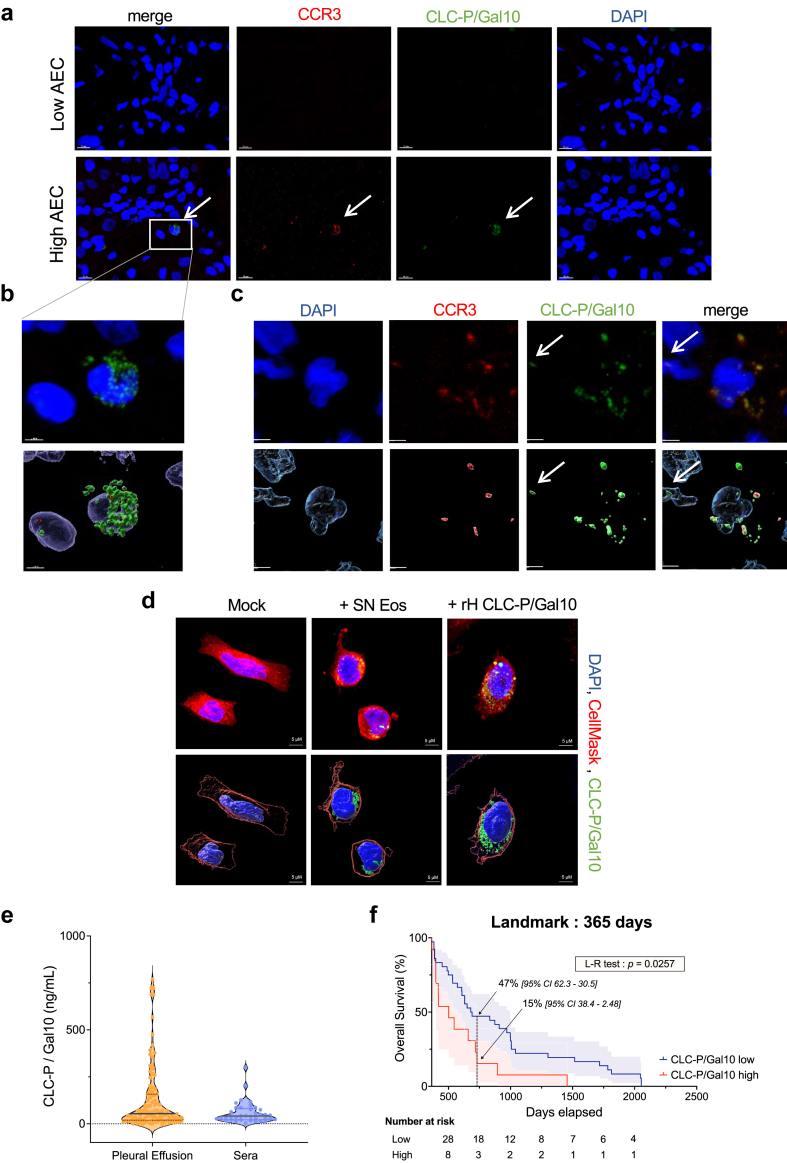
**CLC-P/Gal10 is present in the tumour microenvironment and correlates with poor survival**. **(a)** Histochemical analyses of fixed tumour biopsies from MPM patients with low and high absolute eosinophil counts (AEC). Samples were fluorescently labelled for CCR3 (APC in red) and CLC-P/Gal10 (in green) and DAPI (in blue). Images were acquired using a Zeiss 880 Airyscan Elyra confocal microscope equipped with a x63–1.4 oil immersion objective. Scale bars are 10 μm. **(b)** Representative image of an eosinophil with a characteristic bilobed nucleus (blue) expressing CLC-P/Gal10 (green) analysed with Imaris. **(c)** CLC-P/Gal10 externalized by a degranulating eosinophil (white arrow). **(d)** M14K cells were incubated either with culture medium (mock), 25% (v/v) SN Eos or recombinant h-CLC-P/Gal10 for 48 h. Cells were fixed, labelled for CLC-P/Gal10 (green) and stained with DAPI (blue) and CellMask (red). Images were acquired using a Zeiss 980 Airyscan Elyra confocal microscope equipped with a x63–1.4 oil immersion objective. Representative images and 3D representations of CLC-P/Gal10 interaction with MPM cells were computed with Imaris. **(e)** ELISA titration of CLC-P/Gal10 (in ng/mL) in pleural effusions (n = 79) and sera (n = 37) from MPM patients. **(f)** Overall survival analysis of patients displaying high (>123.8 ng) and low (<123.8 ng) CLC-P/Gal10 levels in MPM pleural fluids.

Because invasion of the tumour microenvironment by eosinophils is infrequent in mesothelioma,^46^ the correlation between CLC-P/Gal10 and chemoresistance may be questionable. To address the clinical relevance of eosinophil-derived CLC-P/Gal10, tumours from patients with mesothelioma were analysed by confocal microscopy. Tumour biopsies from subjects displaying low and high absolute eosinophil counts (AEC) in the peripheral blood (85 and 1019 eosinophils/μL of blood, respectively) were stained with DAPI and fluorescently labelled for CCR3 and CLC-P/Gal10. Confocal imaging highlighted the presence of CCR3-CLC-P/Gal10 double-positive cells characterised by a bilobed nucleus, revealing that eosinophils indeed infiltrated the tumour (Fig. 5a–b). Of note, these eosinophils were nevertheless infrequent (approximately from 0 to 0.5% of cells) (Supplementary Fig. S15), consistent with the literature.^46^ In these patient's biopsies, cell-free granules of CLC-P/Gal10 also directly interacted with CCR3-negative tumour cells (arrow on Fig. 5c). Similarly, human CLC-P/Gal10 produced by recombinant technology or derived from eosinophil supernatant (SN Eos) was internalized by M14K (Fig. 5d) and ZL34 cells (Supplementary Fig. S16).

Collectively, these data thus showed that eosinophils and their cell-derived components were present in the tumour microenvironment and could be internalized by MPM cells.

### The levels of CLC-P/Gal10 in pleural fluids correlate with poor survival

Since eosinophils were poorly abundant in mesothelioma tumours, it was possible that CLC-P/Gal10 originated from the pleural effusions or the peripheral blood. Titration by ELISA indicated that significant amounts of CLC-P/Gal10 were detected in pleural effusions and in the serum of patients with mesothelioma (Fig. 5e; Supplementary Table S5). The CLC-P/Gal10 levels in the serum were lower than but correlated with those in pleural effusions (152.5 ± 21.6 ng/mL *vs.* 58.28 ± 9.48). Using the rpart software, a threshold of 123.8 ng of CLC-P/Gal10 per mL of pleural fluid optimally segregated patients according to overall survival. Multivariate analysis did not highlight another prognosis factor in the cohort, confirming CLC-P/Gal10 as an independent prognosis factor.

Aalen's regression indicated that the correlation of CLC-P/Gal10 high/low groups with survival varied overtime and became significant at one year (Supplementary Fig. S17). Using a landmark approach, Kaplan Meier analysis of the survival time limited to the first year indicated that the CLC-P/Gal10 levels did not correlate with survival (*p* = 0.87, Supplementary Fig. S17). After one year however, the threshold of 123.8 ng CLC-P/Gal10 per mL of pleural effusion identified two cohorts with significantly different overall survival (*p* = 0.0257, Fig. 5f).

### An anti-eosinophilic treatment restores the effectiveness of chemotherapy in mice

**Fig. 6:**
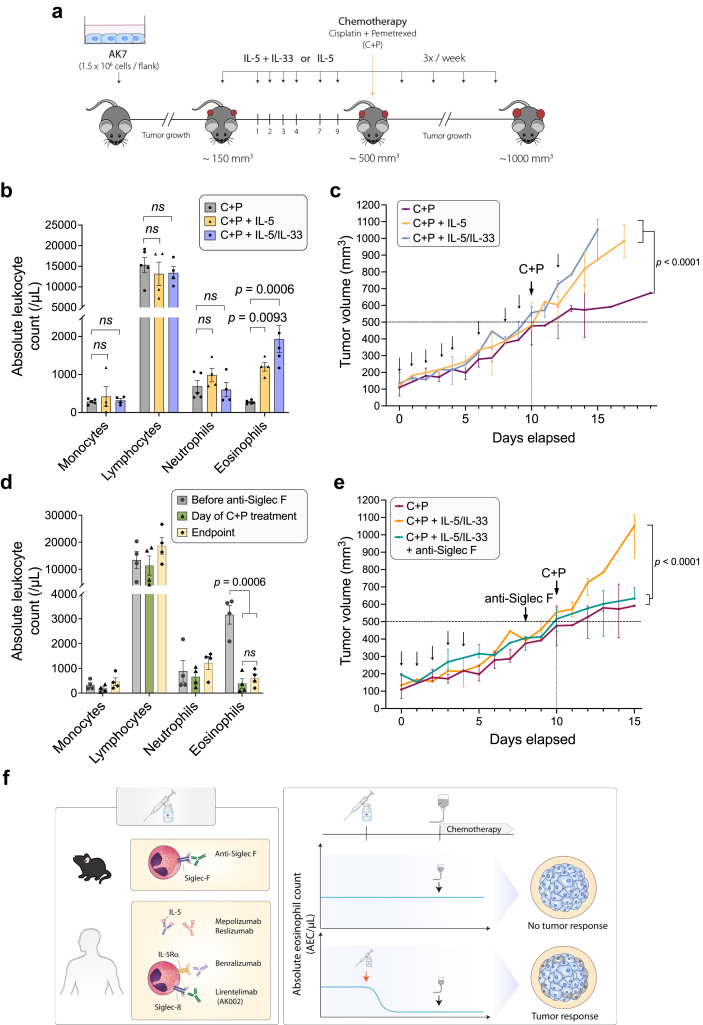
**Peripheral blood eosinophilia inhibits chemotherapy in mice while an anti-eosinophilic treatment restores effectiveness**. **(a)** Experimental design. C57BL/6 mice were implanted subcutaneously with epithelioid AK7 mesothelioma cells (1.5 × 10^6^ cells/flank). When the tumour reached ∼150 mm^3^, eosinophilia was increased with IP injections of IL-5 and/or IL-33 as indicated. When the tumour reached ∼500 mm^3^, mice were given C + P chemotherapy and tumour growth was assessed until tumour reached 1000 mm^3^. **(b)** Absolute counts (number of cells/μL of blood collected from the tail vein) at Day 10 of the different leukocyte populations measured with an hematocytometer just before C + P treatment. **(c)** The tumour volume (mm^3^) was regularly determined by using the hemi-ellipsoid formula (L x H x W x π/6), where L=length, W=width, H=height. (C+P, C+P + IL-5, C+P + IL-5 + IL-33 *n* = 5). **(d)** Eosinophilic mice were injected with anti-Siglec F antibody 2 days prior to chemotherapy administration. Absolute leukocyte counts were measured before (grey) and concomitantly (green) with chemotherapy administration, and at the end of the experiment (yellow). **(e)** Tumour growth was determined as in panel c (C + P + IL-5/IL-33 + anti-Siglec F *n* = 4). Growth curves were constructed based on median and range. Normality of the populations and homogeneity of variances were checked by Shapiro–Wilk and by Levene's test, respectively. Growth curves were compared by using the “growth index” method followed by two-way ANOVA. **(f)** Perspectives for an improved mesothelioma therapy. Based on the mouse model, the reduction of eosinophils is predicted to ameliorate the survival in human mesothelioma.

To investigate the role of eosinophils in response to chemotherapy in a preclinical mouse model, the epithelioid mesothelioma AK7 cell line was inoculated into syngeneic C57BL/6.^47^ At an early stage of tumour growth (∼150 mm^3^), eosinophilia was induced with cytokines IL-5 and/or IL-33^29^^,^^48^ (Fig. 6a). In these experimental settings, the absolute eosinophil counts in the peripheral blood at day 10 significantly increased upon administration of IL-5 or IL-5 + IL-33, while other leukocyte populations (*i.e*., monocytes, lymphocytes and neutrophils) remained unaffected (Fig. 6b). The administration of these two cytokines did not significantly modify tumour growth kinetics (Supplementary Fig. S18) but induced eosinophil infiltration in the tumour, as revealed by MBP staining (Supplementary Fig. S19). When the tumours reached ∼500 mm^3^, C + P chemotherapy reduced tumour growth, as anticipated (Supplementary Fig. S20). In eosinophilic mice however, mesothelioma tumours were unresponsive to an effective dose of C + P (*p* < 0.0001, f^2^ = 1.382, Fig. 6c), indicating that excess of eosinophils impaired the response to chemotherapy.

To investigate the efficacy of an anti-eosinophilic treatment, mice were treated with the anti-Siglec F antibody two days before chemotherapy (D8). A single dose of the neutralizing anti-Siglec F antibody restored the baseline eosinophil counts (Fig. 6d) as well as the response to the C + P regimen (*p* < 0.0001, f^2^ = 3.034, Fig. 6e).

In conclusion, preclinical data in a syngeneic mouse model demonstrated that the excess of peripheral blood eosinophils impaired the response to the C + P chemotherapy. Antibody-mediated ablation of eosinophils restored the efficacy of the C + P regimen, providing preclinical evidence for an improved therapy of mesothelioma based on an anti-eosinophilic treatment.

## Discussion

In mesothelioma, several examples illustrate the involvement of the local microenvironment in tumour growth and response to therapy.^9^^,^^49^^,^^50^ The role of the macroenvironment is by far more controversial although the prognostic impact of systemic inflammation achieves a broader consensus.^23^ Blood inflammatory markers such as total leucocyte count, neutrophil/lymphocyte ratio (NLR) and C-reactive protein (CRP) have been inversely correlated with survival.^50^ Recently, we added an additional layer of complexity by including another cell type of the myeloid lineage. Indeed, retrospective data sets from 230 patients with mesothelioma collected in 3 clinical centres indicate that an excess of peripheral blood eosinophils prior to chemotherapy is associated with worse outcome and quicker relapse.^25^ Since this correlation does not imply causation, we have now investigated the mechanism mediating the interplay between eosinophils and response to chemotherapy. Based on experimental evidence in cell culture and mice, we demonstrate that eosinophil-derived CLC-P/Gal10 promotes resistance to the standard chemotherapy of mesothelioma (*i.e.,* the C + P regimen) and, more importantly, that an anti-eosinophilic treatment allows to improve the therapeutic response in a preclinical mouse model.

This report thus opens new prospects for improved therapeutic options in the most frequent subtype of mesothelioma affecting the pleura. The less prevalent form, sarcomatoid mesothelioma, is mostly unresponsive to chemotherapy and is preferably treated with ICIs. Reflecting these clinical disparities, two cell lines modelling the epithelioid (M14K) and non-epithelioid (ZL34) subtypes display different responses to C + P chemotherapy (Supplementary Figs. S1–S3). Similar differences were also observed in low-passage cultures obtained from mesothelioma tumours representing the three major histological subtypes (Supplementary Figs. S13 and S14). There is thus a spectrum of responses to CLC-P/Gal10, indicating a mechanistic complexity. The behaviour of these cells reveals that response to chemotherapy involves a delicate equilibrium between S phase arrest and apoptosis. Transcriptomic analyses highlight a series of GOs involved in cisplatin and pemetrexed response that are suppressed by the Dif-EOL1 supernatant but also marked differences between the M14K and ZL34 cell lines that may reflect their response to eosinophil-derived factors. It also highlights several unexpected pathways that illustrate the complexity of the mechanisms involved. Further experiments are required to clarify the mechanisms of eosinophil-mediated resistance to chemotherapy, particularly, in the context of primary cells.

The paradigm is even more complicated by the ability of mesothelioma cells to undergo senescence upon chemotherapy.^45^ Platinum based compounds induce extensive genomic lesions through covalent adducts and intra- or inter-strand DNA cross-linking. Consequently, damaged tumour cells undergo senescence to avoid further genomic instability and accumulation of DNA lesions.^51^ Senescence and senescence-associated secretory phenotype (SASP) have been associated with mesothelioma chemoresistance in cell cultures and in patients.^44^^,^^52^^,^^53^ Consistently, our data also show that chemotherapy induced SA-β-gal activity in epithelioid mesothelioma cells. Furthermore, Dif-EOL1 and Eos supernatant increased chemotherapy-induced SA-β-gal activity (Fig. 3, Fig. 4). This indicates that eosinophils may induce mesothelioma chemoresistance by promoting senescence and/or SASP. Interestingly, cell cycle analysis revealed that the reduction of DNA fragmentation promoted by eosinophil supernatant was compensated by S phase blockade (Supplementary Fig. S3), which could be consistent with senescence.

Experimental evidence obtained in this study demonstrates that CLC-P/Gal10 is a mediator of eosinophil-mediated chemoresistance. The CLC-P/Gal10 protein is a small hydrophobic polypeptide of 142 amino acids that interacts with a lysophospholipase inhibitor and promotes lysophosphatidylcholine hydrolysis.^54^^,^^55^ In its insoluble form, crystalized CLC-P has been recognized as a classical hallmark of eosinophilic inflammation in tissues and body fluids.^56^^,^^57^ These Charcot-Leyden crystals result from non-covalent aggregation of Gal10 leading to highly insoluble and remarkably stable aggregates. We have been unable to identify these Charcot-Leyden crystal structures in the series of tumour biopsies available from the CHU tumour bank. Instead, evidence suggests that CLC-P/Gal10 granules may be externalized to sites of inflammation (Fig. 5a–c), possibly via extracellular vesicles (EVs) or eosinophil extracellular traps (EETs).^58^, ^59^, ^60^ Accordingly, the time-lapse imaging reveals that Dif-EOL1 degranulate CFSE-labelled components into M14K mesothelioma cells (Fig. 3a). Confocal imaging further reveals that primary eosinophils degranulate and form EETs in presence of M14K cells (Fig. 3b). It is thus possible that, besides CLC-P/Gal10, other eosinophil components such as EETs are involved in chemoresistance. Importantly, eosinophil-derived and recombinant CLC-P/Gal10 enter the cytoplasm of mesothelioma cells (Fig. 5d). Collectively, these evidences support a model postulating that CLC-P/Gal10 containing granules produced by eosinophils affect mesothelioma cells through a juxtracrine route (as illustrated by the arrow on Fig. 5c). It is also possible that CLC-P/Gal10 is produced by eosinophils in the circulation and/or accumulates in the pleural fluid (Fig. 5f), affecting mesothelioma cells via a paracrine mode. In fact, the juxta- and paracrine mechanisms are not mutually exclusive and may affect mesothelioma chemosensitivity. This notwithstanding, a direct interaction between mesothelioma cells and eosinophils is unlikely due to their infrequent infiltration in tumours (Fig. 5 and Supplementary Fig. S15). It remains nevertheless possible that eosinophils are short-lived and degranulate after tumour invasion upon contact with mesothelioma cells. It should be noted that our results do not support a paracrine way of CLC-P/Gal10 secretion stimulated by MPM cells (Supplementary Fig. S12). Although a significant number of pleural fluids were analysed for CLC-P/Gal10 levels (Supplementary Table S5),^61^^,^^62^ the study should be extended to independent cohorts.

The role of CLC-P/Gal10 in mediating chemoresistance is demonstrated by deprivation of eosinophil-conditioned supernatant and by complementation of culture medium with CLC-P/Gal10 protein. CLC-P/Gal10 may thus be a novel therapeutic target to reduce chemoresistance in mesothelioma. In this context, camelid single domain anti- CLC-P/Gal10 antibodies with therapeutic value in Th2-type inflammatory airway diseases may be of particular interest.^58^ In addition to a CLC-P/Gal10-specific therapy, it may be useful to evaluate alternative options targeting eosinophils. However, particular attention should be paid to the indirect effects of anti-eosinophilic treatment as eosinophils release cytotoxic factors (granzyme, MBP, ECP and EDN) that can destroy tumours.^29^ Eosinophil-derived cytokines IL-12 and IL-10 decrease metastasis by enhancing E-cadherin expression on tumour cells. Eosinophils also release IFN-γ, which acts in an autocrine manner or in combination with CD8+ T cells. Furthermore, TNF-α and IFN-γ-activated eosinophils polarize macrophages towards an anti-tumorigenic M1 phenotype. Therefore, a therapy targeting eosinophils may also favour tumour growth and be detrimental to patients’ outcome. However, there is to our knowledge no evidence supporting the anti-tumorigenic role of eosinophils in mesothelioma. Importantly, our data shows that 2 central cytokines modulating eosinophil fate (*i.e.*, IL-5 and IL-33) do not significantly modify tumour growth kinetics in a mouse model of mesothelioma (Supplementary Fig. S18). In contrast, an anti-eosinophil treatment improves the effectiveness of chemotherapy in a preclinical model (Fig. 6), thereby supporting further investigations in patients with mesothelioma. In this perspective, it should be mentioned that a dose of C + P was selected to stabilize but not completely clear the tumour in the preclinical model (Fig. 6 and Supplementary Fig. S20). Note that a limitation of the mouse model is that the ortholog of the human CLC-P/Gal10 has not been clearly identified in mice. However, the Supplementary Fig. S19 shows that MBP, a typical eosinophil-associated protein, localizes in AK7 tumours in mice, particularly when the blood eosinophil level is high. This observation thus indicates that eosinophils and eosinophil-associated proteins infiltrate MPM tumours in the mouse model.

Although our experimental design mirrors the partial response to platinum-based regimen in patients with mesothelioma, we are aware that the pharmacokinetics of the chemotherapeutic compounds are different in mice and humans. This trivial statement is becoming a particularly hot topic for targeted immunotherapies. In a concept of metronomic therapy that would better preserve the host immunity, further experiments are needed to broaden the conclusions in clinical settings. Notwithstanding, there is concordance between evidence obtained in cell cultures, mouse models and clinical datasets that support the detrimental role of eosinophils in mesothelioma and particularly their CLC-P/Gal10 content. A strategy aimed at reducing the eosinophil counts just prior to chemotherapy is therefore predicted to provide a clinical benefit to patients with mesothelioma. The paradigm is however complexified by the different responses measured in low-passage primary cells, which may reflect the disparity observed in patients. In cell culture, resistance to chemotherapy was evaluated through Annexin V/PI labelling, DNA fragmentation and S phase blockage. In mice, the resistance to chemotherapy was defined by the progression of the tumour growth despite C + P administration. In patients, the response to treatment is based on the RECIST criteria of complete response, partial response, stable disease and progressive disease.^25^^,^^63^^,^^64^ The interpretation of the cell culture and mouse models combined with patients’ data sets nevertheless converge to the main conclusion.^25^

There is a number of strategies for reducing the number of eosinophils. Although this question has not been specifically studied, it is still unclear why methylprednisolone, a glucocorticoid frequently combined with chemotherapy to limit inflammation, does not improve the effectiveness of chemotherapy. Among different hypotheses, it is possible that the kinetics of administration is inappropriate because methylprednisolone doses are given concurrently with chemotherapy. The anti-inflammatory activity of methylprednisolone may also affect other immune cell types that modulate patient's response. It is also conceivable that similar approaches focusing on identical targets may have different outcomes as illustrated by the non-overlapping effects of PD-1 and PDL-1 immunotherapies. In the same line, therapies targeting eosinophil-modulating cytokines such as IL-5, IL-33 or their receptors may have more specific effects than broad anti-inflammatory drugs, such as methylprednisolone. A series of monoclonal antibodies interacting with these cytokines, such as the anti-IL-5 antibodies mepolizumab and reslizumab, and the anti-IL5-Rα antibody benralizumab, are readily available for clinical use to treat eosinophil-associated diseases.^65^ Based on the findings reported here and promising observations in eosinophilic gastritis/duodenitis,^66^ we propose that Siglec-8, the human ortholog of mouse Siglec-F which induce eosinophil apoptosis, may be a priority candidate to target in mesothelioma therapy.

## Contributors

M.W. and L.W. wrote the original manuscript and designed figures (visualization). M.W., M.H. and L.W. designed the experiments. M.W., L.H., M.H. and C.B. performed the experiments and acquired the data. M.W., A.F. and M.G. performed mice experiments. M.W., A.F. and L.V.M designed bioinformatic analyses. S.D. performed all ELISA experiments. Y.B. designed and performed patients’ survival analyses. M.W., M.H., C.B. and L.W. analysed and interpreted the data. A.F., L.H., M.G., L.V.M., R.T., M.J., D.J., V.H., R.L., B.D., A.S., E.W. and C.B. reviewed the manuscript. M.W., L.W. and M.H. directly accessed and verified the underlying data reported in the manuscript. All authors read and approved the final version of the manuscript.

## Data sharing statement

Publicly available datasets analysed in this study can be found on the library: https://www.ncbi.nlm.nih.gov/bioproject/PRJNA1049163 accessed on 12 December 2023. The datasets generated and analysed during the current study are available from the corresponding authors on reasonable request. All datasets used for figures are provided as Supplementary Materials.

## Declaration of interests

The authors declare no conflict of interest in the scope of this work. Beyond the scope of this study, RL declares consultancy fees from GSK and AstraZeneca, and grants from GSK AstraZeneca, Sanofi and Chiesi.

## Appendix A Supplementary data

Supplementary Table S5

Reagents validation
