# Supplementary material for: The impact of Charcot-Leyden Crystal protein on mesothelioma chemotherapy: targeting eosinophils for enhanced chemosensitivity
Source: eBioMedicine. 2024 Oct 30;109:105418. doi: 10.1016/j.ebiom.2024.105418 (PMC11550731; doi:10.1016/j.ebiom.2024.105418)
Supplement: Supplementary Figures [file mmc1.pdf]

The impact of Charcot-Leyden Crystal protein on mesothelioma chemotherapy: targeting eosinophils for enhanced chemosensitivity

Authors: Mégane Willems [1,*], Malik Hamaidia [1,*], Alexis Fontaine [1], Mélanie Grégoire [1], Louise Halkin [1], Lea Vilanova Mañá [1], Roxane Terres [1], Majeed Jamakhani [1], Sophie Deshayes [2], Yves Brostaux [3], Vincent Heinen [4], Renaud Louis [4], Bernard Duysinx [4], Didier Jean [5], Eric Wasielewski [6], Arnaud Scherpereel [6], Christophe Blanquart [2] and Luc Willems [1]

Affiliations:
[1] Molecular and Cellular Epigenetics (GIGA) and Molecular Biology (TERRA), University of Liege, Liege & Gembloux, Belgium
[2] Institut National de la Santé et de la Recherche Médicale (INSERM) U1232 Centre de Recherche en Cancérologie et Immunologie Nantes Angers (CRCINA), Nantes, France.
[3] Modelisation and development, Gembloux Agro-Bio Tech, University of Liege, Gembloux, Belgium
[4] Department of Pneumology (University Hospital of Liege), Liege, Belgium
[5] Centre de Recherche des Cordeliers (INSERM), Sorbonne Université (Université de Paris), Functional Genomics of Solid Tumors, Paris, France
[6] Department of Pneumology and Thoracic Oncology (CHU Lille) and INSERM U1189 (ONCOTHAI), Lille, France

[*] co-first authors

Correspondence: Luc Willems, Molecular and Cellular Epigenetics, Interdisciplinary Cluster for Applied Genoproteomics (GIGA), University of Liège, B34, 1 avenue de l'Hôpital, 4000 Sart-Tilman Liège, Belgium. Phone: 32.81.622152; Email: luc.willems@uliege.be.

Table of content

Fig. S1. EOL1-conditioned medium affects the apoptotic response of non-epithelioid ZL34 cells to chemotherapy 3

Fig. S2. EOL1-conditioned medium affects late apoptosis of M14K and ZL34 cells in response to chemotherapy 4

Fig. S3. Compared to M14K, non-epithelioid ZL34 cells preferably undergo S phase arrest in presence of C+P 5

Fig. S4. Co-administration of Dif-EOL1 and C+P does not affect apoptosis 6

Fig. S5. Transcriptomic profiles of ZL34 cells in response to Dif-EOL1 supernatant 7

Fig. S6 Gene ontology enrichment analysis of M14K CP *vs.* mock 8

Fig. S7 Gene ontology enrichment analysis of ZL34 CP *vs.* mock 9

Fig. S8 Gene ontology enrichment analysis of M14K CP+ SN Dif-EOL1 *vs.* CP 10

Fig. S9 Gene ontology enrichment analysis of ZL34 CP + SN Dif-EOL1 *vs.* CP 11

Fig. S10. Expression of CLC-P/Gal10 increases upon differentiation of EOL1 into Dif-EOL1 cells 12

Fig. S11. C+P treatment does not modify CLC-P/Gal10 secretion by primary eosinophils ... 13

Fig. S12. Supernatant of MPM cells does not significantly affect CLC-P/Gal10 expression by primary eosinophils 14

Fig. S13. Low-passage primary cells from mesothelioma patients show different responses to cisplatin and pemetrexed 15

Fig. S14. Low passage primary MPM cultures exhibit different cell cycle proportion response to CLC-P/Gal10 16

Fig. S15. Immunofluorescence of tumour biopsies from MPM patients 17

Fig. S16. ZL34 cells internalize CLC-P/Gal10 18

Fig. S17. The CLC-P/Gal10 levels in pleural fluids of MPM patients do not correlate with survival during the first-year post-diagnosis 19

Fig. S18. Cytokine-induced blood eosinophilia does not influence tumour growth in C57BL/6 20

Fig. S19. Expression of MBP is increased in tumours of eosinophilic mice 21

Fig. S20. The cisplatin+pemetrexed regimen reduces tumour growth in C57BL/6 mice 22

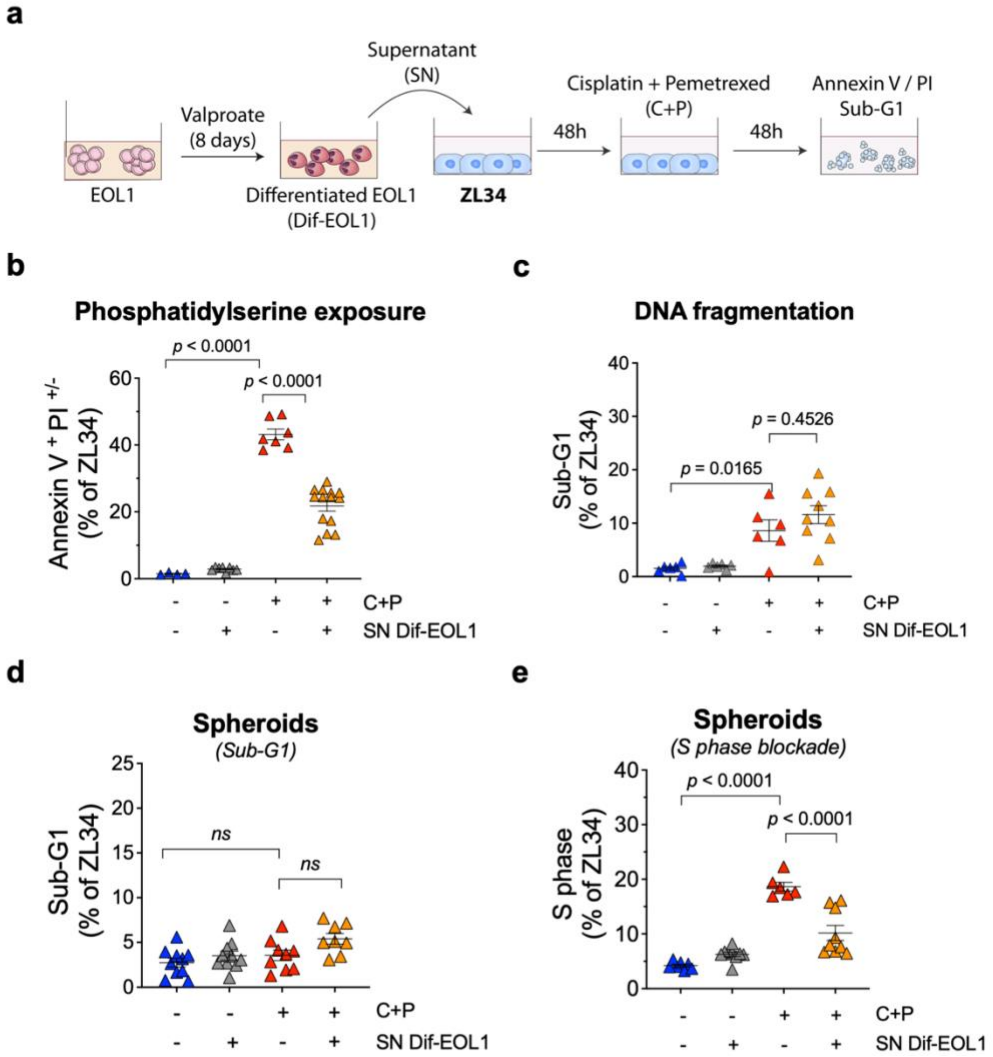

Fig. S1. EOL1-conditioned medium affects the apoptotic response of non-epithelioid ZL34 cells to chemotherapy

(a) Experimental design. EOL1 progenitors were differentiated in eosinophils (Dif-EOL1) with valproate for 8 days. Supernatant from differentiated cells (SN Dif-EOL1) was added at 25% v/v to the medium of ZL34 cell cultures for 48 hours. After treatment with 10 µM cisplatin and 10 µM pemetrexed (C+P) for 48 hours, the rates of apoptosis were quantified by Annexin V/PI labelling and cell cycle analysis (Sub-G1). **(b)** Percentages of apoptotic ZL34 cells evaluated by flow cytometry analysis of annexin V/PI labelled cells. **(c)** After ethanol permeabilization and PI staining, the proportion of cells with fragmented DNA (*i.e.*, Sub-G1) was determined by flow cytometry. **(d)** ZL34 cells were cultured for 72 hours in a 96-well plate coated with DMEM-agarose 1.5% in presence of SN Dif-EOL1 and then treated with C+P for 2 days. The proportion of ZL34 cells with fragmented DNA (*i.e.*, Sub-G1) was analysed after spheroid dissociation, cell permeabilization and propidium iodide staining. **(e)** S phase blockade in ZL34 spheroids. Data are expressed as means +/- SD, each dot representing an independent test. Normality of the data distribution was analysed by Shapiro-Wilk and equality of the variances was determined by Brown-Forsythe. The variance of the means was compared by one-way ANOVA followed by Tukey's multiple comparison test.

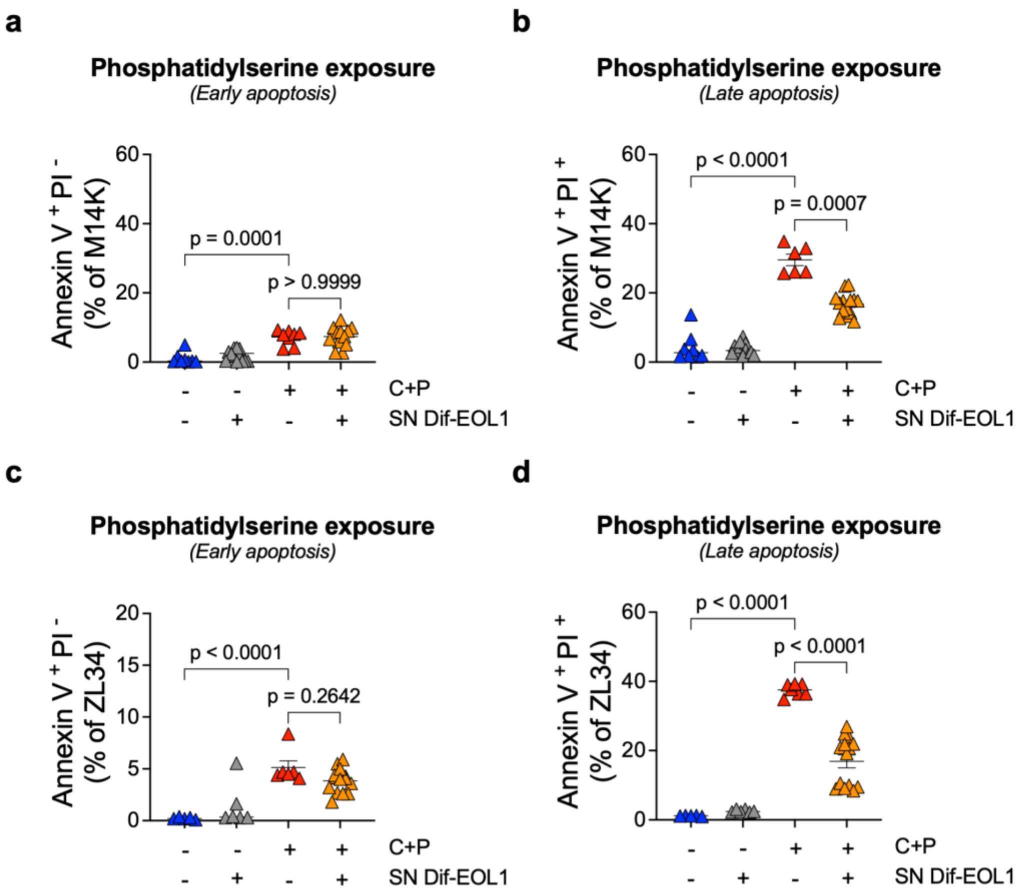

Fig. S2. EOL1-conditioned medium affects late apoptosis of M14K and ZL34 cells in response to chemotherapy

After annexin V/PI labelling, the proportion of M14K **(a,b)** and ZL34 **(c,d)** cells in early (*i.e.*, Annexin V^+ PI^-) **(a,c)** and late apoptosis (*i.e.*, Annexin V^+ PI^+) **(b,d)** were determined by flow cytometry. Data is expressed as means +/- SD, each point representing an independent test. Normality of the data distribution was analysed by Shapiro-Wilk and equality of the variances was determined by Brown-Forsythe. Variance of the means was compared by one-way ANOVA followed by Tukey's multiple comparison test.

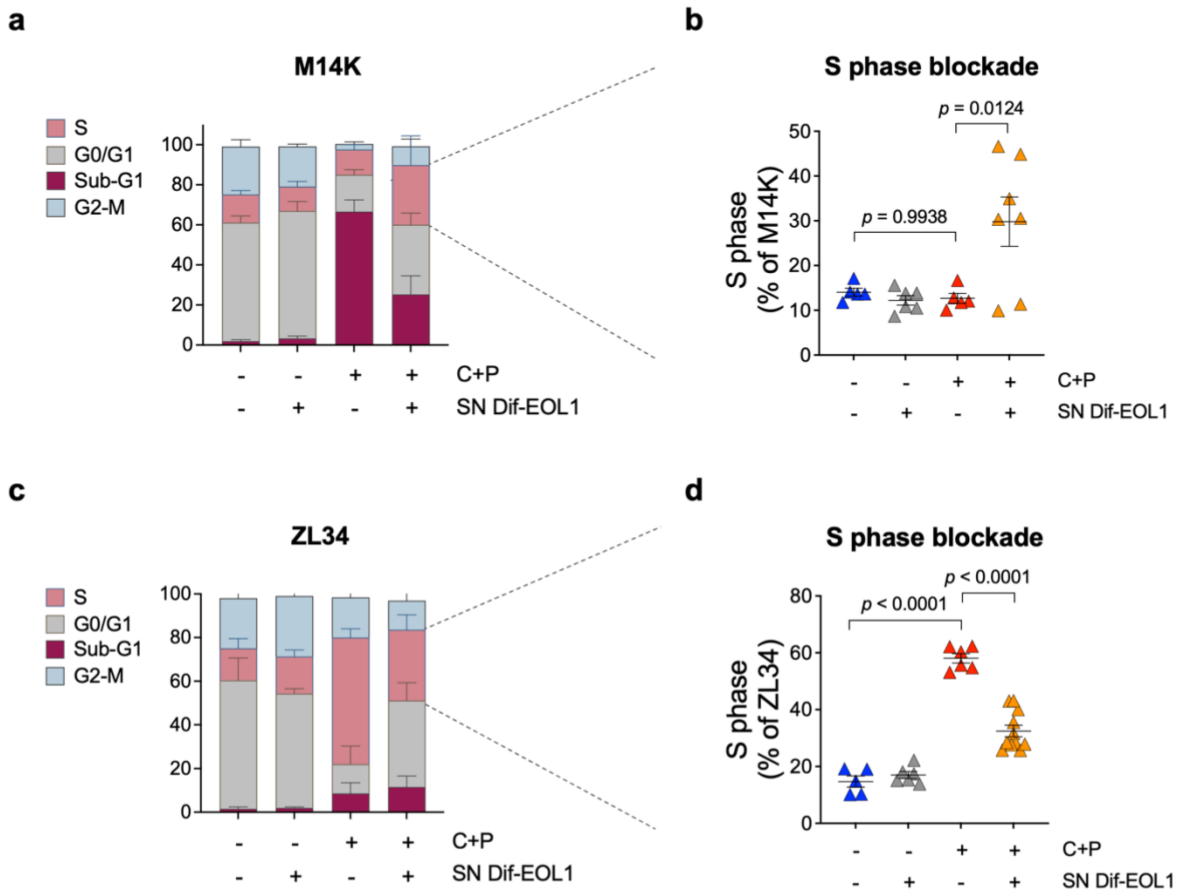

Fig. S3. Compared to M14K, non-epithelioid ZL34 cells preferably undergo S phase arrest in presence of C+P
Representative cell cycle distributions of M14K **(a)** and ZL34 **(c)** cells. The proportion of cells in each phase of the cell cycle (Sub-G1, G0, S and G2-M) in the different conditions are indicated. Statistical analysis of S-phase blockade in M14K **(b)** and ZL34 **(d)**. Data is expressed as means +/- SD, each point representing an independent test. Normality of the data distribution was analysed by Shapiro-Wilk and equality of the variances was determined by Brown-Forsythe. Variance of the means was compared by one-way ANOVA followed by Tukey’s multiple comparison test.

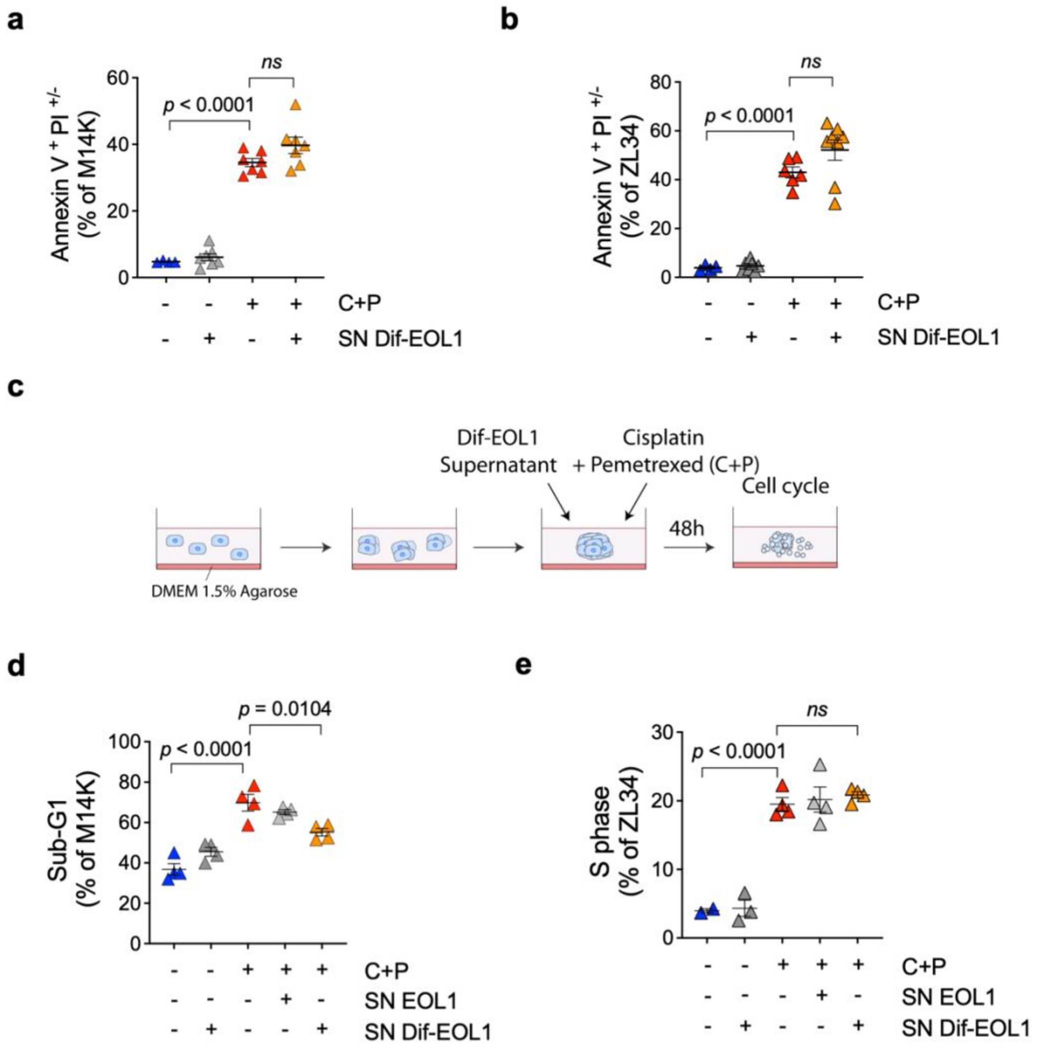

Fig. S4. Co-administration of Dif-EOL1 and C+P does not affect apoptosis
MPM cells were cultured in presence of differentiated EOL-1 supernatant (SN Dif-EOL1) at 25% v/v, 10µM cisplatin and 10 µM pemetrexed (C+P) for 48 hours. After labelling with Annexin V/PI, apoptosis was evaluated by flow cytometry. Apoptotic rates in M14K **(a)** and ZL34 **(b)** cells. **(c)** MPM cells were cultured in a 96-well plate coated with DMEM-agarose 1.5% for 72 hours. Spheroids were then cultured for 48 hours in presence or absence of SN Dif-EOL1 and 10µM C+P. After spheroid dissociation, ethanol permeabilization and propidium iodide staining, the proportion of M14K cells with fragmented DNA (*i.e.*, Sub-G1) **(d)** and ZL34 cells in S phase **(e)** were analysed by flow cytometry. Data is expressed as means +/- SD, each dot representing an independent test. Normality of the data distribution was analysed by Shapiro-Wilk and equality of the variances was determined by Brown-Forsythe. The variance of the means was compared by one-way ANOVA followed by Tukey's multiple comparison test.

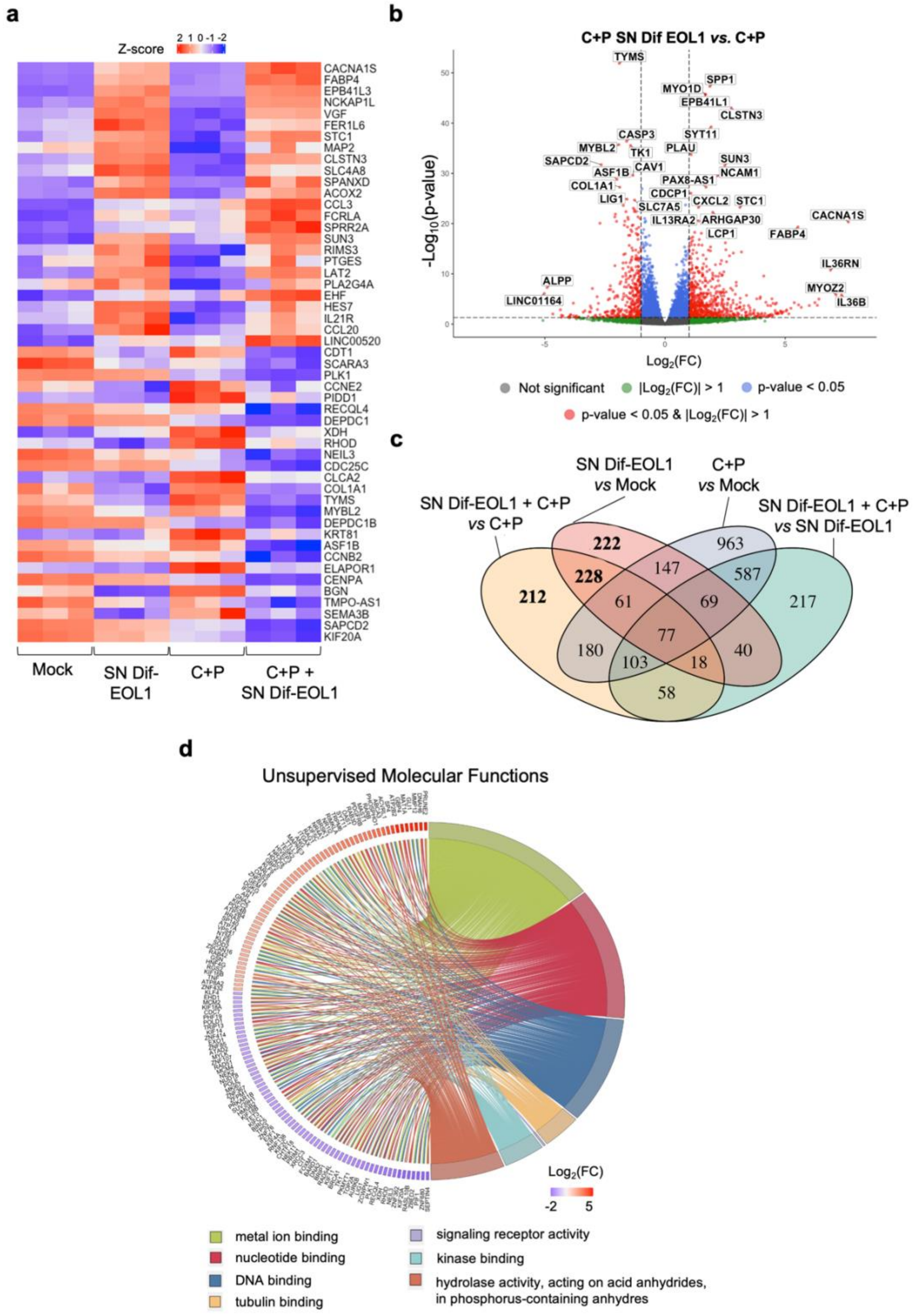

Fig. S5. Transcriptomic profiles of ZL34 cells in response to Dif-EOL1 supernatant
(a) Unsupervised heatmap of the top 25 up- and down-regulated most significant genes deduced from 3 independent experiments. **(b)** Volcano plot of DEGs in M14K treated with C+P in presence of mock or conditioned medium of differentiated EOL1 (SN Dif-EOL1). DEGs $|Log_2(FC)| > 1$ and p-adj ≤ 0.05 are coloured in red. **(c)** Venn diagram of significant DEGs in the different conditions. Numbers of genes impacted by SN Dif-EOL1 are in bold. **(d)** Representative chord diagram of the most significative pathways affected in GO Molecular Functions (GO:MF) in conditions C+P+SN Dif-EOL1 and C+P. Pathways (right half of the diagram) are linked to the genes (on the left side) according to their Log_2.

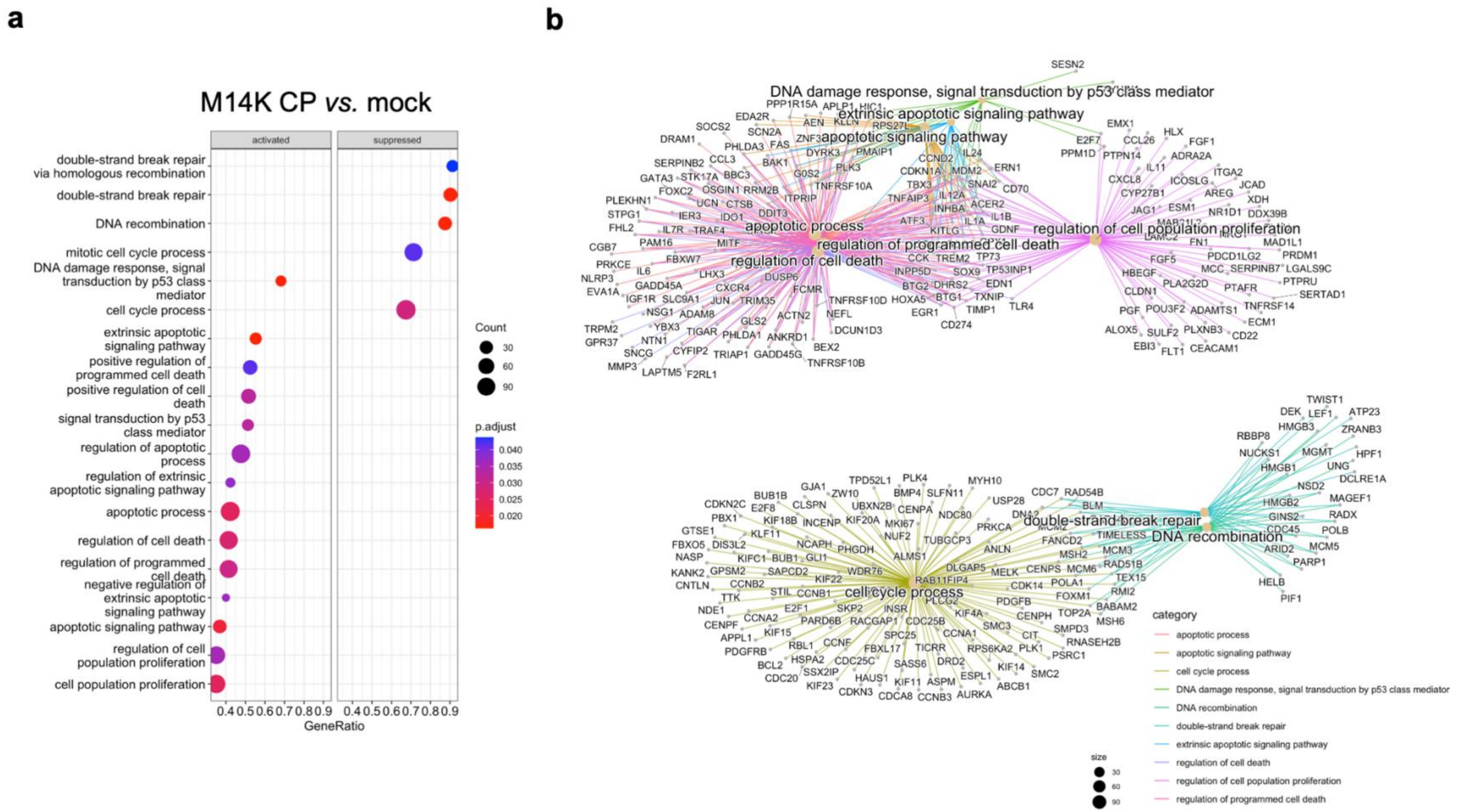

Fig. S6. Gene ontology enrichment analysis of M14K CP *vs.* mock

(a) Dot plot of the significant pathways related to cisplatin+pemetrexed response as results of gene ontology (GO) of the gene set enrichment analysis (GSEA) of M14K CP *vs.* mock. Dot size represents the number of genes belonging to each pathway. The colour gradient is related to the level of significance, adjusted with the Benjamini-Hochberg method. The box on the left collects activated pathways, while the box on the right the suppressed ones. **(b)** The gene concept network reports the enriched pathways in the list of differentially expressed genes (DEGs) resulting from the comparison CP *vs.* mock. Individual genes are represented in grey dots, while GOs are represented in category colour. The size of GO points indicates the number of significantly enriched genes belonging to the specific GO.

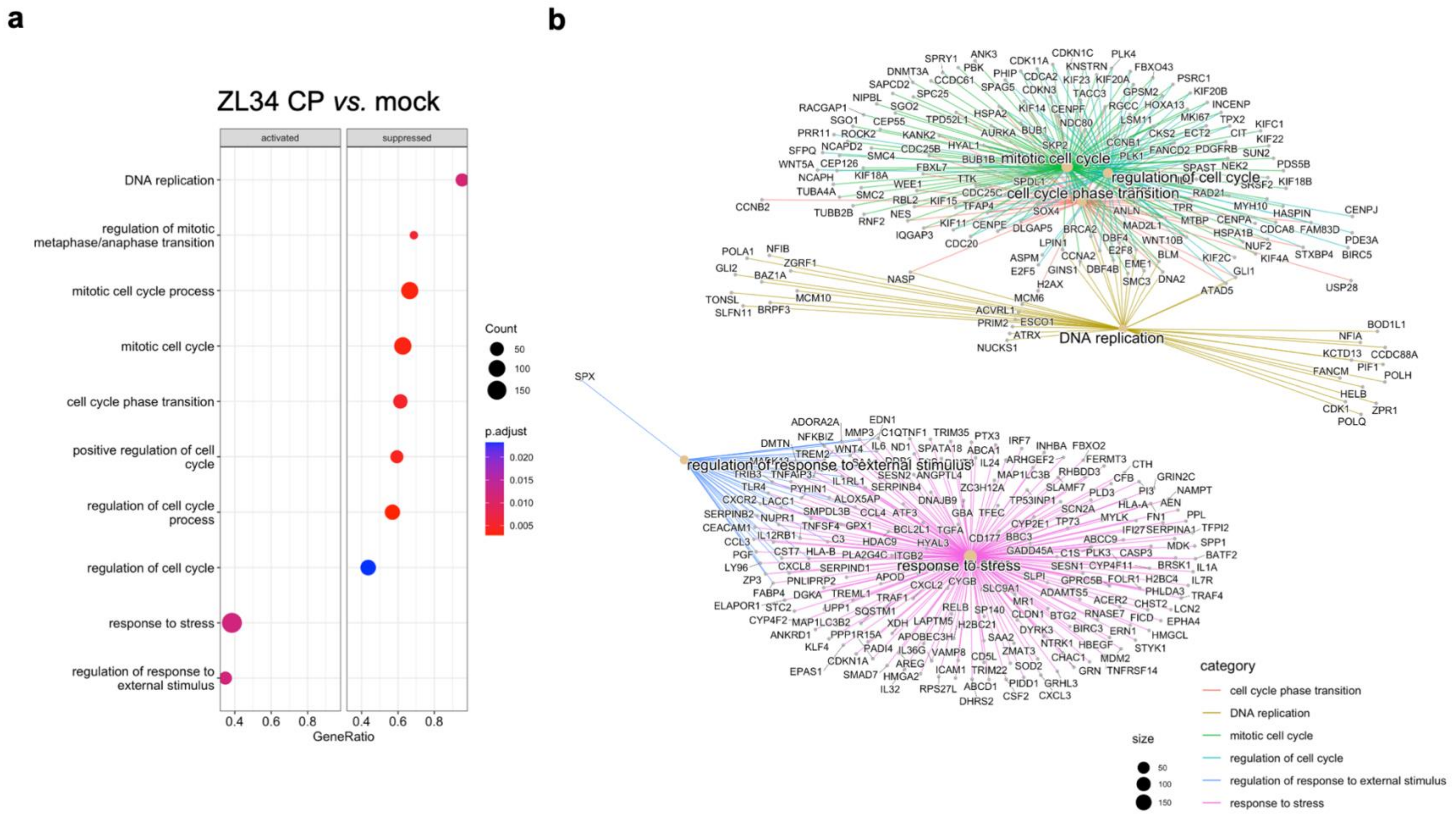

Fig. S7. Gene ontology enrichment analysis of ZL34 CP *vs.* mock

(a) Dot plot of the significant pathways related to cisplatin+pemetrexed response as results of gene ontology (GO) of the gene set enrichment analysis (GSEA) of ZL34 CP *vs.* mock. Dot size represents the number of genes belonging to each pathway. The colour gradient is related to the level of significance, adjusted with the Benjamini-Hochberg method. The box on the left collects activated pathways, while the box on the right the suppressed ones. **(b)** The gene concept network reports the enriched pathways in the list of differentially expressed genes (DEGs) resulting from the comparison CP *vs.* mock. Individual genes are represented in grey dots, while GOs are represented in category colour. The size of GO points indicates the number of significantly enriched genes belonging to the specific GO.

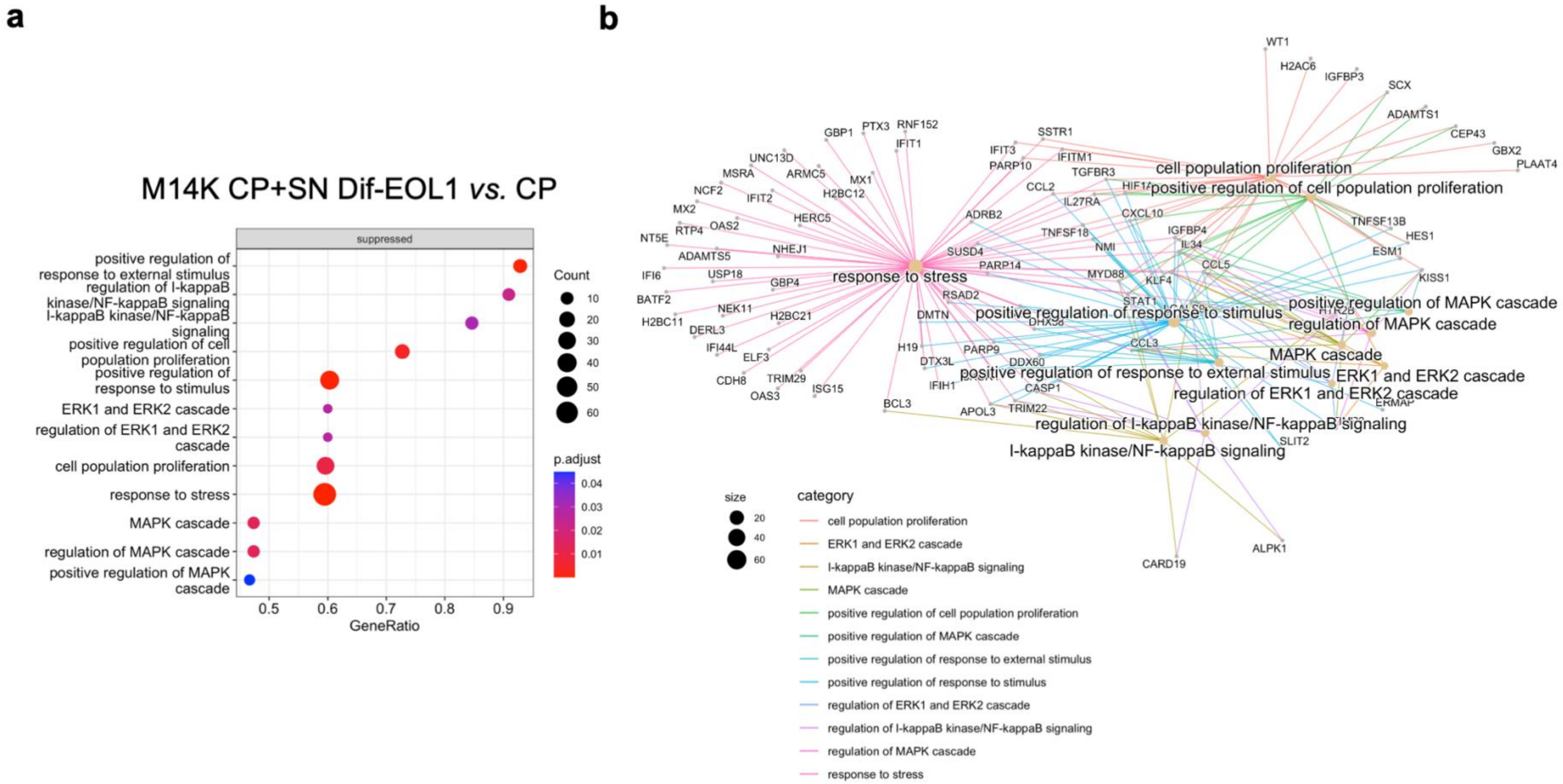

Fig. S8. Gene ontology enrichment analysis of M14K CP+ SN Dif-EOL1 *vs.* CP

(a) Dot plot of the significant pathways related to cisplatin+pemetrexed response and resistance, as results of gene ontology (GO) of the gene set enrichment analysis (GSEA) of M14K CP + SN Dif-EOL1 *vs.* M14K CP. Dot size represents the number of genes belonging to each pathway. The colour gradient is related to the level of significance, adjusted with the Benjamini-Hochberg method. **(b)** The gene concept network reports the enriched pathways in the list of differentially expressed genes (DEGs) resulting from the comparison CP + SN Dif-EOL1 *vs.* CP. Individual genes are represented in grey dots, while GOs are represented in category colour. The size of GO points indicates the number of significantly enriched genes belonging to the specific GO.

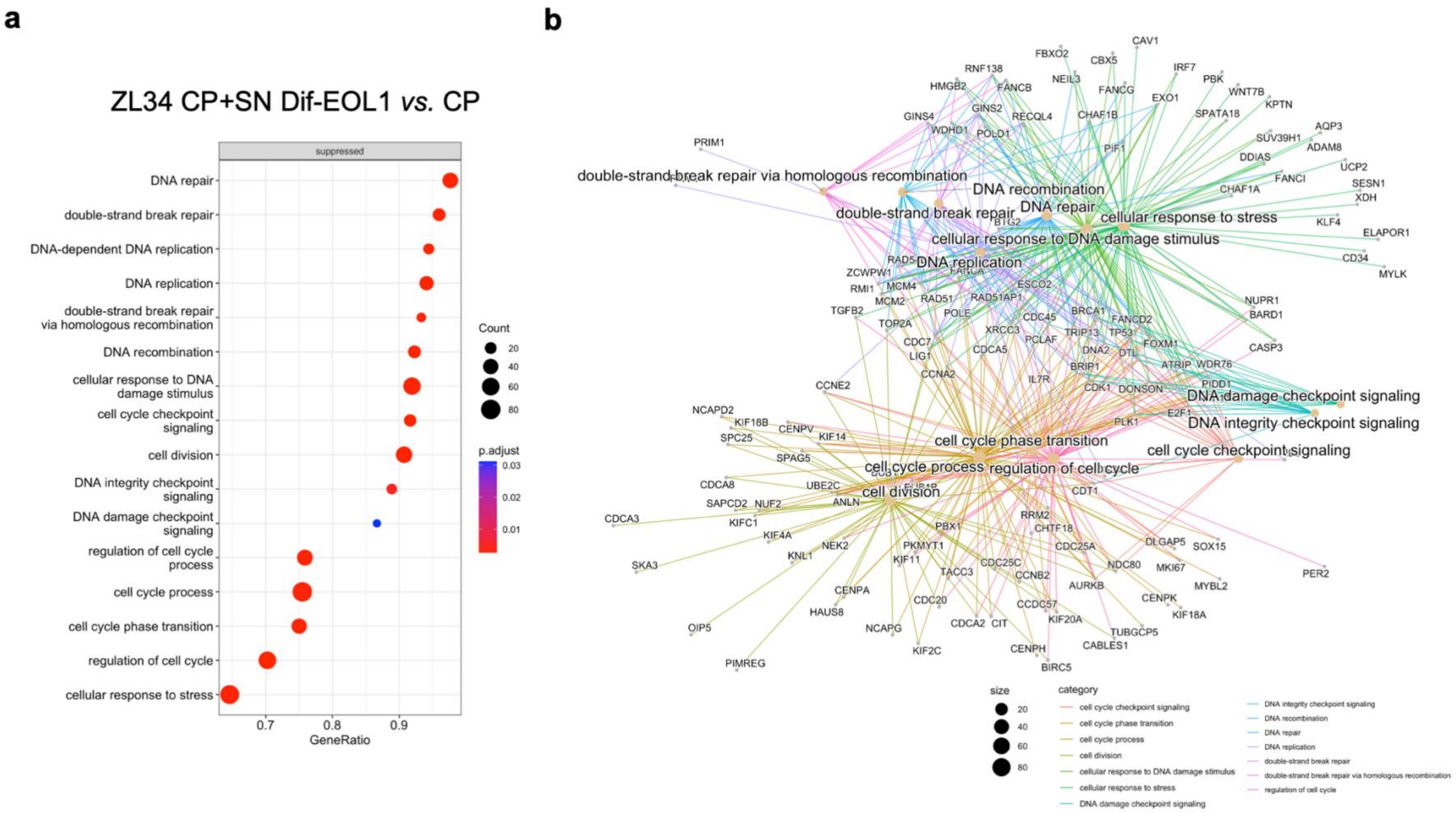

Fig. S9. Gene ontology enrichment analysis of ZL34 CP + SN Dif-EOL1 *vs.* CP

(a) Dot plot of the significant pathways related to cisplatin+pemetrexed response and resistance, as results of gene ontology (GO) of the gene set enrichment analysis (GSEA) of ZL34 CP + SN Dif-EOL1 *vs.* ZL34 CP. Dot size represents the number of genes belonging to each pathway. The colour gradient is related to the level of significance, adjusted with the Benjamini-Hochberg method. **(b)** The gene concept network reports the enriched pathways in the list of differentially expressed genes (DEGs) resulting from the comparison CP + SN Dif-EOL1 *vs.* CP. Individual genes are represented in grey dots, while GOs are represented in category colour. The size of GO points indicates the number of significantly enriched genes belonging to the specific GO.

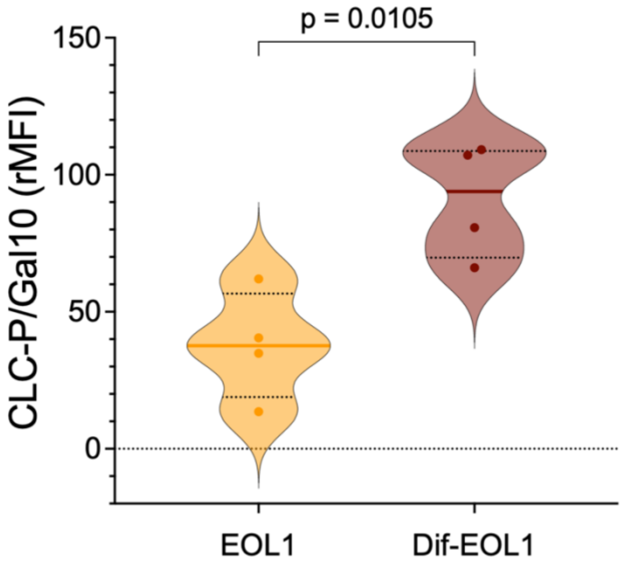

Fig. S10. Expression of CLC-P/Gal10 increases upon differentiation of EOL1 into Dif-EOL1 cells

EOL1 were differentiated (Dif-EOL1) for 8 days with valproate 2 µM. After 8 days, both EOL1 progenitors and Dif-EOL1 were collected, fixed, permeabilized, stained for CLC-P/Gal10 and analysed by flow cytometry. The relative median intensity (rMFI) corresponds to the ratio of intensities associated with CLC-P/Gal10 compared to control isotype. Normality of the population was checked with Shapiro-Wilk and variance of the means was compared by unpaired t-test using Welch's correction.

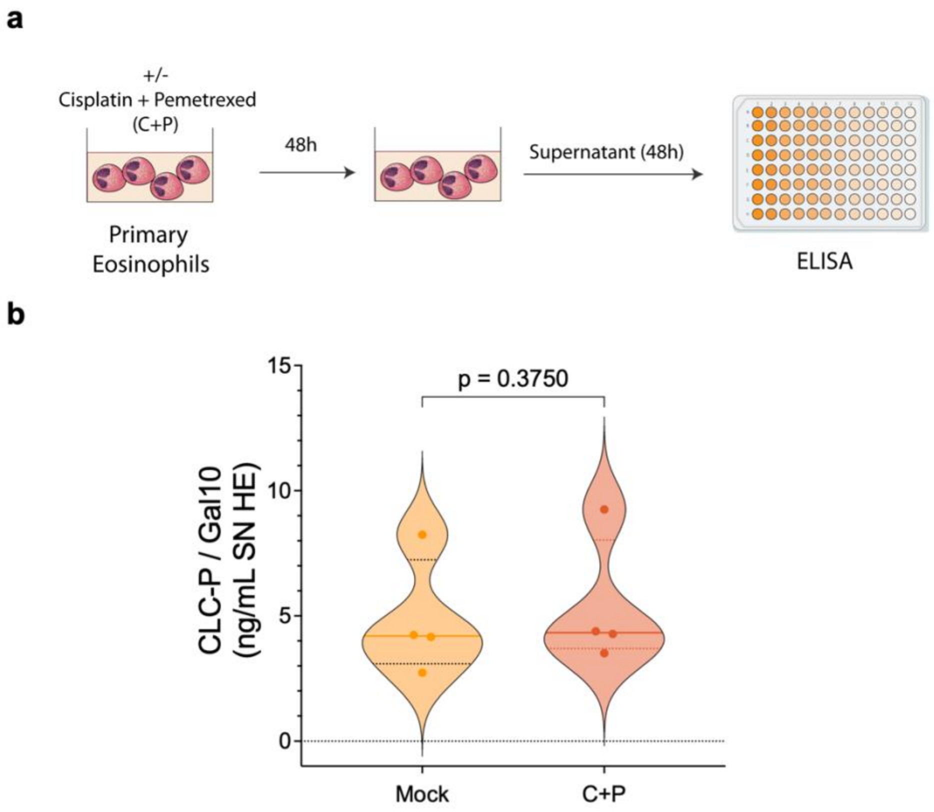

Fig. S11. C+P treatment does not modify CLC-P/Gal10 secretion by primary eosinophils
(a) Experimental design. Primary eosinophils were isolated by Ficoll gradient centrifugation and purified with CCR3-magnetic beads. Eosinophils were then cultured in presence of mock or C+P (10 µM cisplatin and 10 µM pemetrexed) for 48 hours. **(b)** The concentration of CLC-P/Gal10 (in ng/mL of eosinophil supernatant) was quantified by ELISA. Normality was checked by Shapiro-Wilk and variance of the means was compared with Wilcoxon matched-pairs signed rank test.

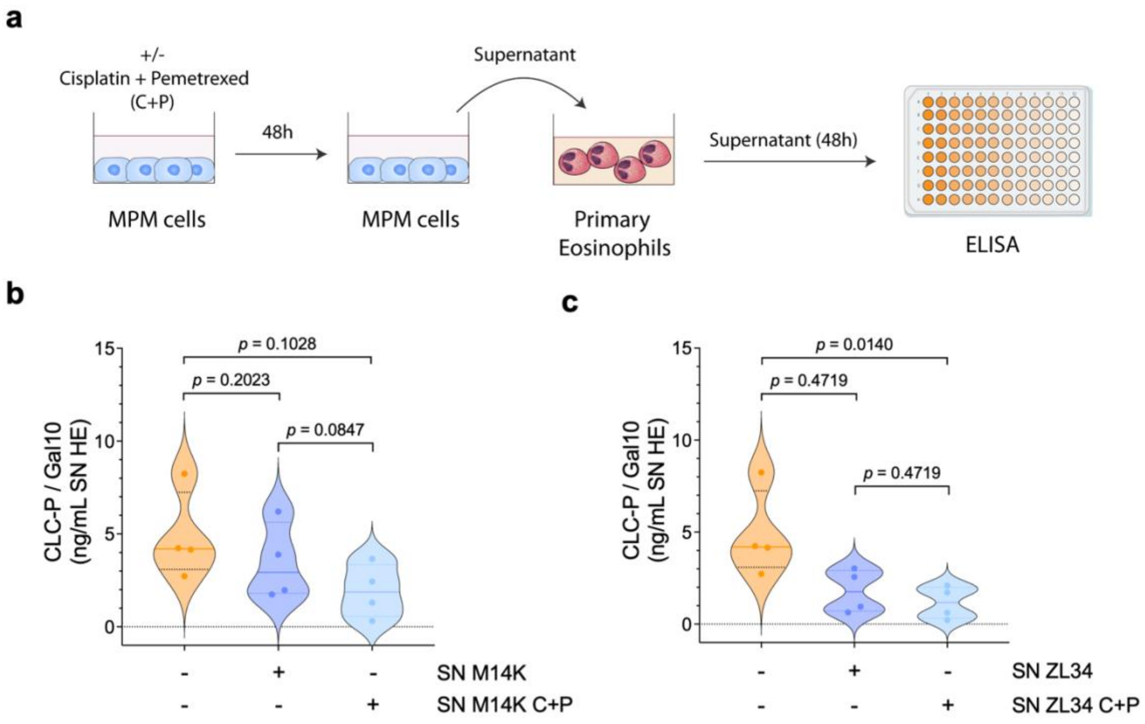

Fig. S12. Supernatant of MPM cells does not significantly affect CLC-P/Gal10 expression by primary eosinophils

(a) Experimental design. M14K and ZL34 cells were cultured in presence or not of cisplatin and pemetrexed for 48 hours. These supernatants were added to primary eosinophils for 48 hours and the concentration of CLC-P/Gal10 (ng/mL) was quantified by ELISA. Normality of the populations was checked by Shapiro-Wilk and equality of the variances were determined by Brown-Forsythe and Welch. Variance of the means was compared by one-way ANOVA followed by Dunn's multiple comparison test.

a

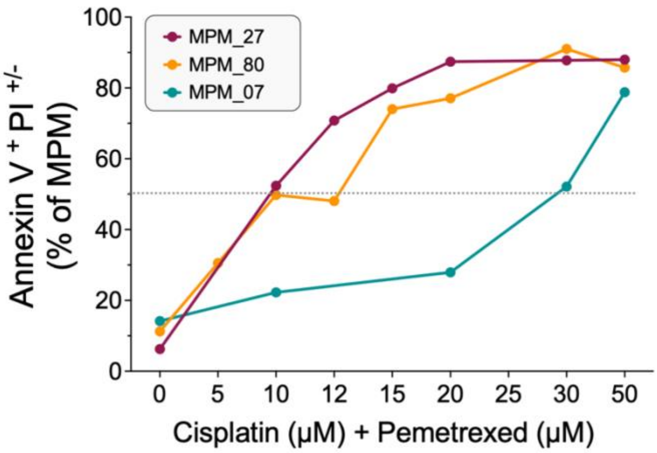

b

Cell line	Sex *(patient)*	Histology	E-score *(RNA-Seq)*	S-score *(RNA-Seq)*	Mutations							IC50
					CDKN2A	TERT	TP53	BAP1	SETD2	NF2	LATS2	
**MPM_07**	M	Epithelioid	1	0	D	WT	WT	M	M	WT	WT	30 µM
**MPM_80**	M	Sarcomatoid	0,06	0,94	D	M	WT	WT	WT	M	WT	10 µM
**MPM_27**	F	Biphasic	0,40	0,60	D	WT	WT	WT	WT	D	WT	10 µM

Fig. S13. Low-passage primary cells from mesothelioma patients show different responses to cisplatin and pemetrexed
(a) Three low-passage cultures established from patients' tumours (#07, #80, and #27) were incubated in presence of cisplatin and pemetrexed at different concentrations (*i.e.*, 0, 10, 15, 20, 25, 30 and 50 µM) for 48 hours. Apoptotic rates were determined by flow cytometry after Annexin V and propidium iodide (PI) staining. **(b)** Histological characteristics and mutational profile of the cell cultures. D: deleted; WT: wild-type; M: mutated; E-score: Epithelioid score; S-score: Sarcomatoid score. E-score and S-score were established by RNA-sequencing [1,2]. The IC50 corresponds to the concentration of C+P inducing 50% apoptosis in these primary cells.

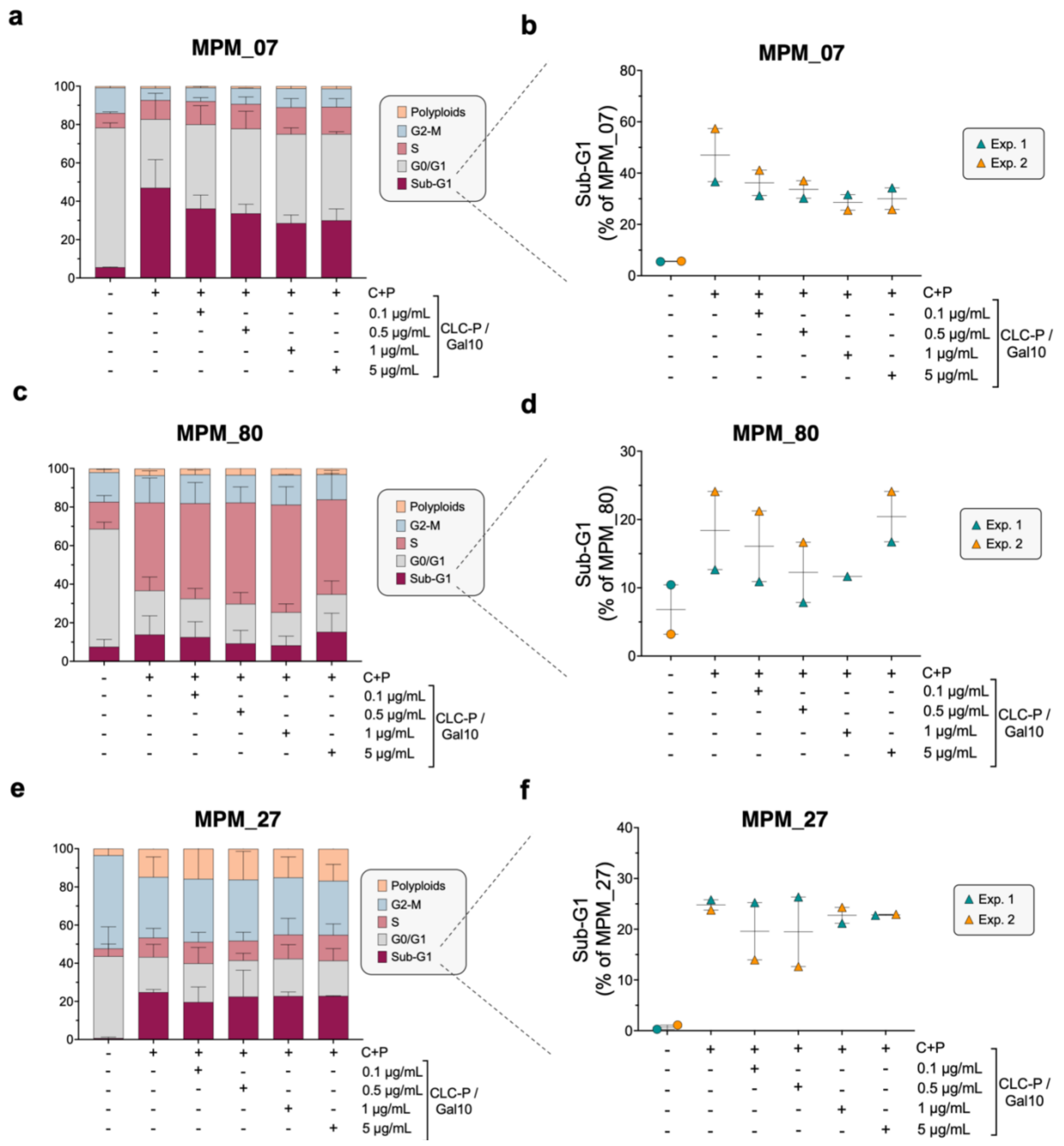

Fig. S14. **Low passage primary MPM cultures exhibit different cell cycle proportion response to CLC-P/Gal10**

Cell cultures from patients #07 (epithelioid), #80 (sarcomatoid) and #27 (biphasic) [1,2] were cultured in presence of CLC-P/Gal10 at 0.1; 0.5; 1 or 5 µg/ml for 48 hours. Then, cells were treated for 48 hours with 10 µM C+P (MPM_80 and MPM_27) or 30 µM C+P (MPM_07). Representative cell cycle distributions of MPM_07 **(a)**, MPM_80 **(c)**, and MPM_27 **(e)**. The proportion of cells in each phase of the cell cycle (Sub-G1, G0, S, G2-M and polyploids) in the different conditions are indicated. **(b, d, f)** Respective analysis of the proportion of cells in Sub-G1. Data is expressed as medians ± 95% CI, each point representing an independent test (Exp. 1 in blue, Exp. 2 in orange).

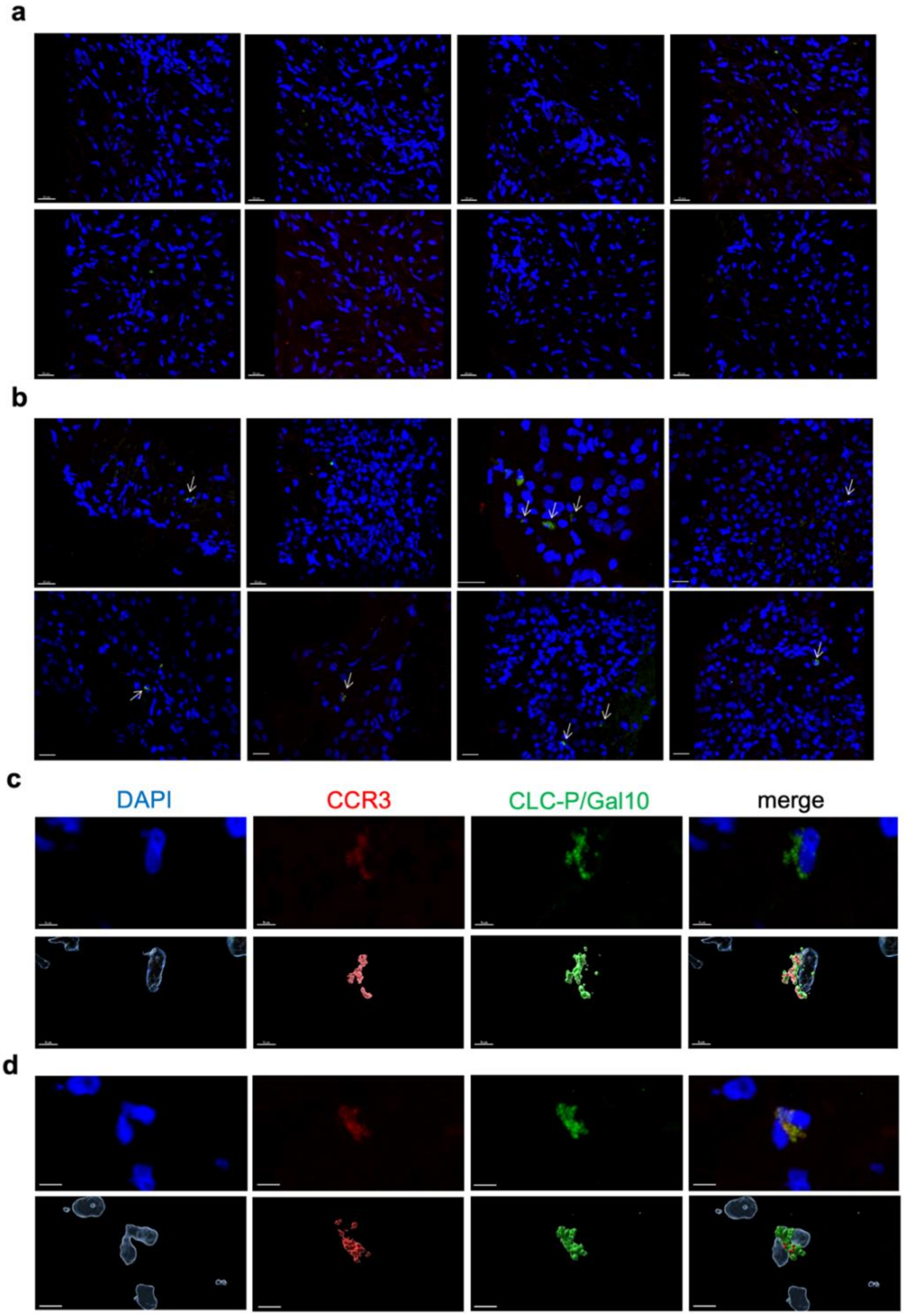

Fig. S15. Immunofluorescence of tumour biopsies from MPM patients

Sections of 4 µm from formalin fixed-tumour biopsies from MPM patient with low **(a)** and high **(b)** absolute eosinophil count (AEC) were stained with DAPI (blue), labelled with anti-CCR3 antibody conjugated with APC (red) and with anti-CLC-P/Gal10 antibody combined with an AlexaFluor488 conjugate (green). Images were acquired using a Zeiss 880 Airyscan Elyra confocal microscope equipped with a x63-1.4 oil immersion objective. The average number of eosinophils infiltrating MPM biopsies (indicated by whit arrows) was estimated on 8 different zones of the same slice. A total of 0 to 17 eosinophils were found in the two tumours, among a total of 3,000 cells per tumour. **(c, d)** Representative images of an eosinophil within the tumour were generated using Imaris. Scale bars represent a length of 3 µm.

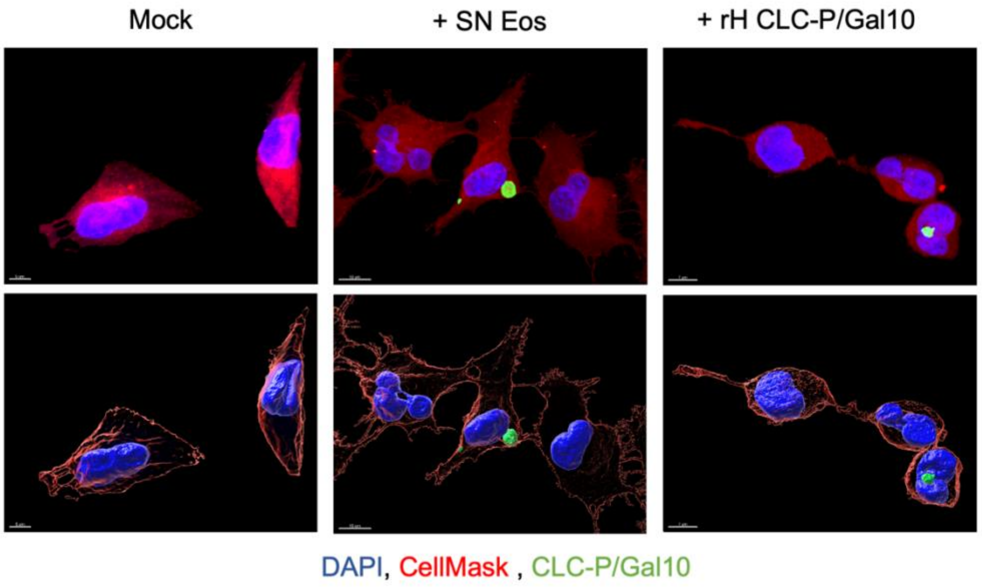

Fig. S16. ZL34 cells internalize CLC-P/Gal10
MPM cells were incubated or not (mock) with SN Eos or recombinant h-CLC-P/Gal10 for 48 hours before staining with DAPI (blue), CellMask (red) and CLC-P/Gal10 (green). Images were acquired using a Zeiss 980 Airyscan Elyra confocal microscope equipped with a x63-1.4 oil immersion objective. Representative images were computed with Imaris.

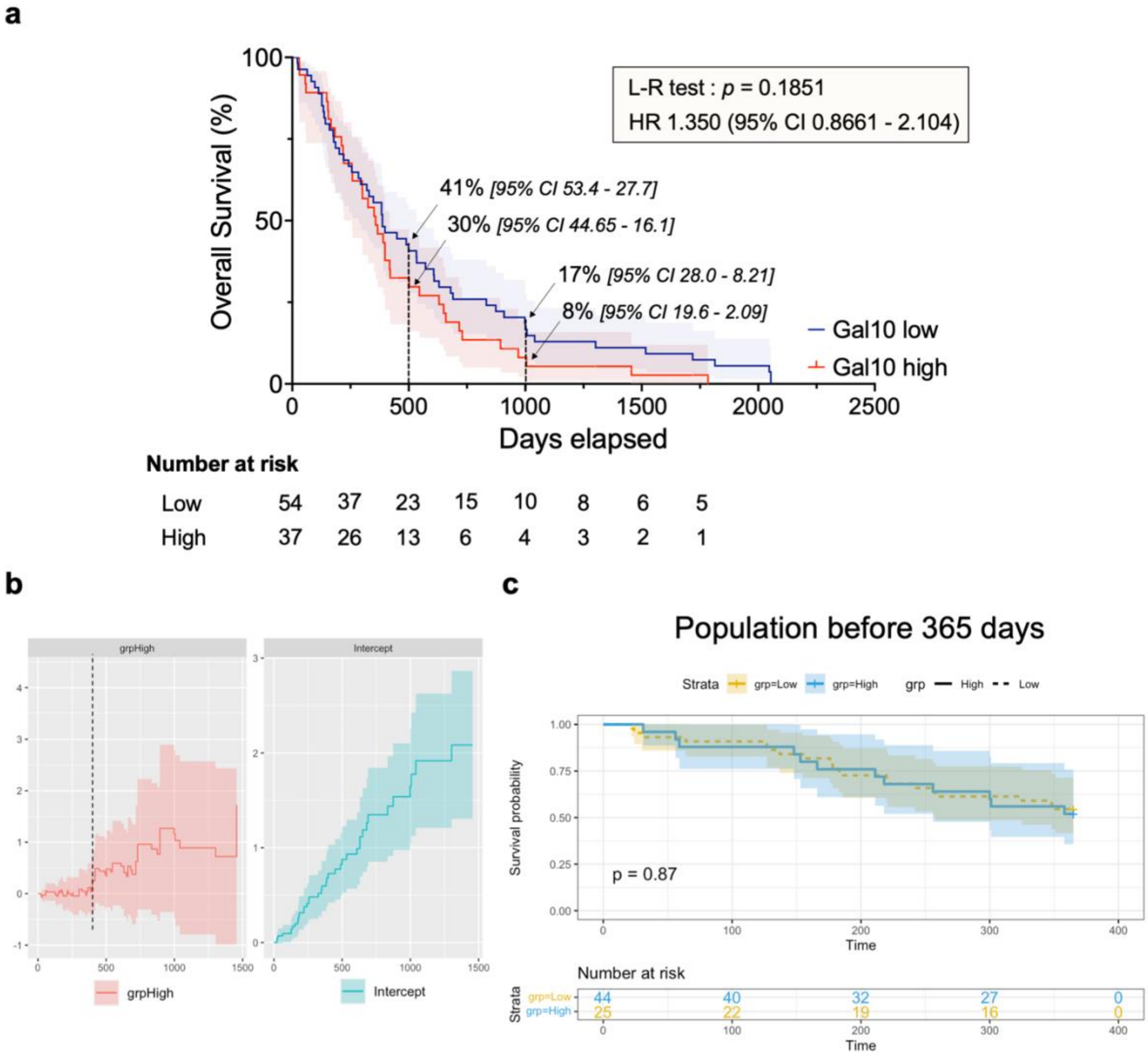

Fig. S17. The CLC-P/Gal10 levels in pleural fluids of MPM patients do not correlate with survival during the first-year post-diagnosis

(a) Correlation of CLC-P/Gal10 in pleural fluids with duration of survival. **(b)** Aalen additive regression model conducted to assess the consistency of the effects of CLC-P/Gal10 groups found with rpart on survival over time. **(c)** Effect of the CLC-P/Gal10 levels on the first-year survival of MPM patients.
L-R test: Log-Rank test; HR: Hazard ratio; CI: Confidence Interval; Grp: group; grpHigh: patients with CLC-P/Gal10 $\geq$ 128.3 ng/mL.

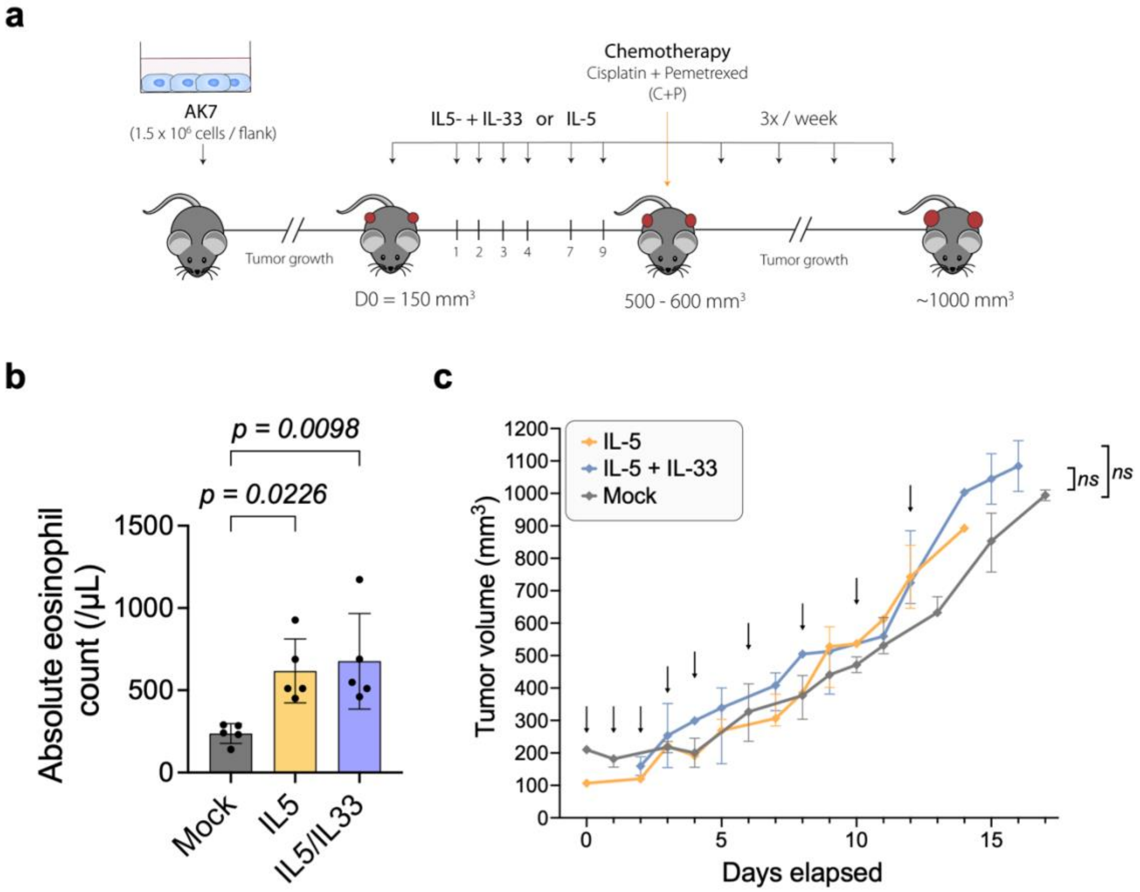

Fig. S18. Cytokine-induced blood eosinophilia does not influence tumour growth in C57BL/6
(a) C57BL/6 mice were inoculated subcutaneously in both flanks with syngeneic AK7 cells. When the tumour reached 200 mm^3, mice were injected intraperitoneally with IL-5 (5 ng /gBW) and IL-33 (20 ng/gBW) as indicated by the arrows. **(b)** Absolute eosinophil counts were determined with an hematocytometer when the tumour reached 500 mm^3, before chemotherapy administration. Normality of the populations was checked by Shapiro-Wilk and homogeneity of the variances was evaluated by Brown-Forsythe. Variance of the means were compared by one-way ANOVA followed by Tukey's multiple comparison test. **(c)** Kinetics of the tumour volume (in mm^3). The tumour volume was regularly determined by using the hemi-ellipsoid formula (L x l x W x π/6), where L=length, W=width, H=height. Growth curves were constructed based on median and range. Normality of the populations was checked by Shapiro-Wilk and homogeneity of the variances was assessed with the Levene's test. Growth curves were compared by using the "growth index" method followed by two-way ANOVA.

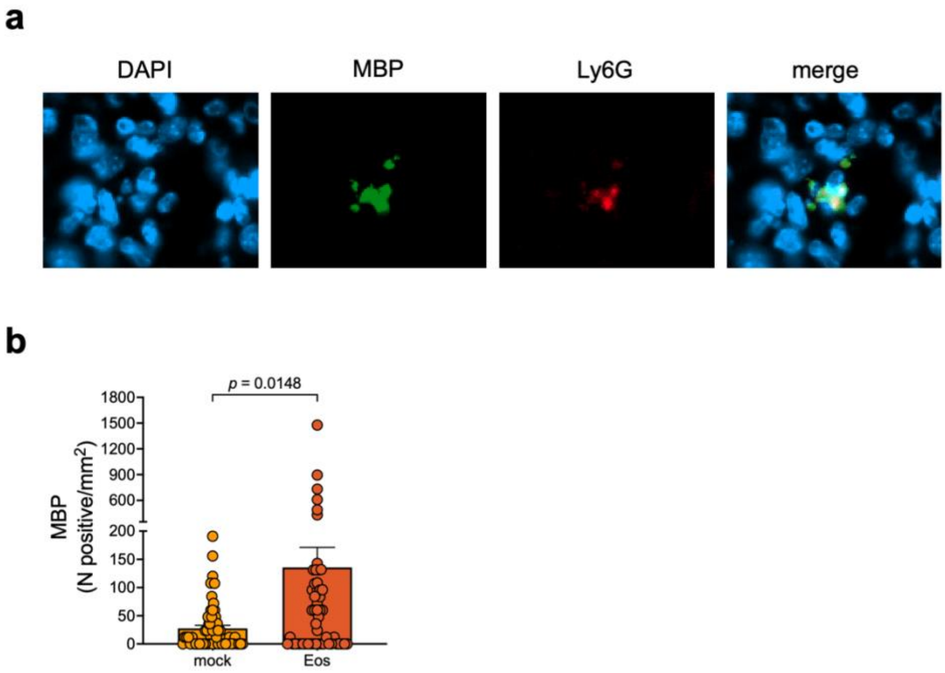

Fig. S19. Expression of MBP is increased in tumours of eosinophilic mice

(a) Representative image of cells co-expressing the major basic protein (MBP) and lysin 6G (Ly6G) within a mesothelioma tumour. After staining with DAPI (blue), formalin-fixed sections were labelled with antibodies specific for the major basic protein (MBP; AlexaFluor 488 in green) and lysine 6G (Ly6G; AlexaFluor 467 in red). Images were acquired using a Zeiss 880 Airyscan Elyra confocal microscope equipped with a x63-1.4 oil immersion objective. **(b)** The average number of Ly6G and MBP positive events was estimated on 15 different zones of the same slice.

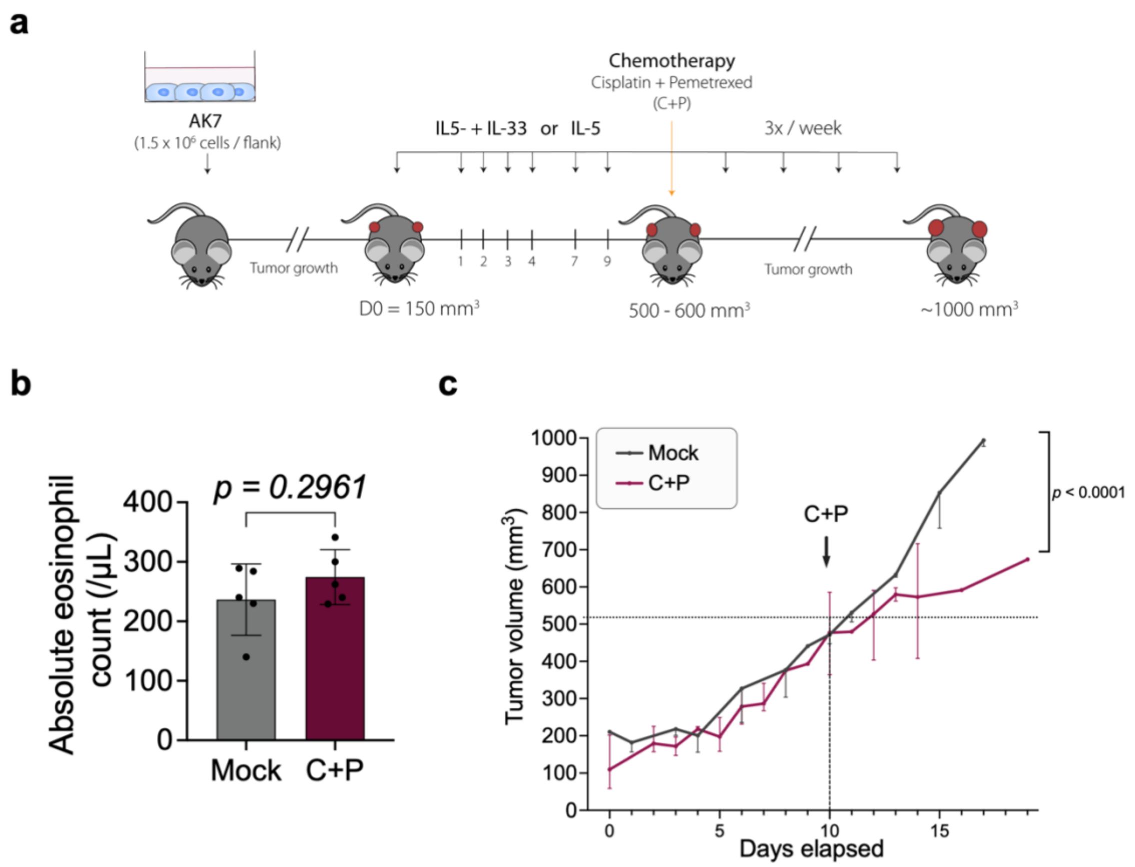

Fig. S20. The cisplatin+pemetrexed regimen reduces tumour growth in C57BL/6 mice
(a) C57BL/6 mice were inoculated subcutaneously with syngeneic AK7 cells. When the tumour reached 500 mm^3, mice were injected with cisplatin (6 µg /gBW) and pemetrexed (150 µg/gBW). (Mock, C+P *n* = 5 mice) **(b)** Absolute eosinophil counts (number of cells / µL of blood) measured with an hematocytometer just before C+P treatment. **(c)** The tumour volume was regularly determined by using the hemi-ellipsoid formula (L x l x W x π/6), where L=length, W=width, H=height. Growth curves were constructed based on median and range. Normality of the populations was checked by Shapiro-Wilk and homogeneity of the variances was assessed with the Levene's test. Growth curves were compared by using the "growth index" method followed by t-test.

References

1 Quetel L, Meiller C, Assié J, Blum Y, Imbeaud S, Montagne F *et al.* Genetic alterations of malignant pleural mesothelioma: association with tumor heterogeneity and overall survival. *Mol Oncol* 2020; 14: 1207–1223.

2 Blum Y, Meiller C, Quetel L, Elarouci N, Ayadi M, Tashtanbaeva D *et al.* Dissecting heterogeneity in malignant pleural mesothelioma through histo-molecular gradients for clinical applications. *Nat Commun* 2019; 10. doi:10.1038/s41467-019-09307-6.
